# Sustainable Food Waste Management in Anaerobic Digesters: Prediction of the Organic Load Impact by Metagenome-Scale Metabolic Modeling

**DOI:** 10.1021/acs.est.4c11180

**Published:** 2025-03-24

**Authors:** Esteban Orellana, Guido Zampieri, Nicola De Bernardini, Leandro D. Guerrero, Leonardo Erijman, Stefano Campanaro, Laura Treu

**Affiliations:** 1 Department of Biology, University of Padua, Via U. Bassi 58/B, Padua 35131, Italy; 2 Instituto de Investigaciones en Ingeniería Genética y Biología Molecular “Dr Héctor N. Torres” (INGEBI-CONICET), Vuelta de Obligado, 2490, Buenos Aires C1428ADN, Argentina; 3 Departamento de Fisiología, Biología Molecular y Celular, Facultad de Ciencias Exactas y Naturales, Universidad de Buenos Aires, Intendente Güiraldes, 2160, Buenos Aires C1428EGA, Argentina

**Keywords:** metabolic modeling, flux balance analysis, anaerobic codigestion, extracellular enzymes, microbial metabolic exchanges, metagenomics

## Abstract

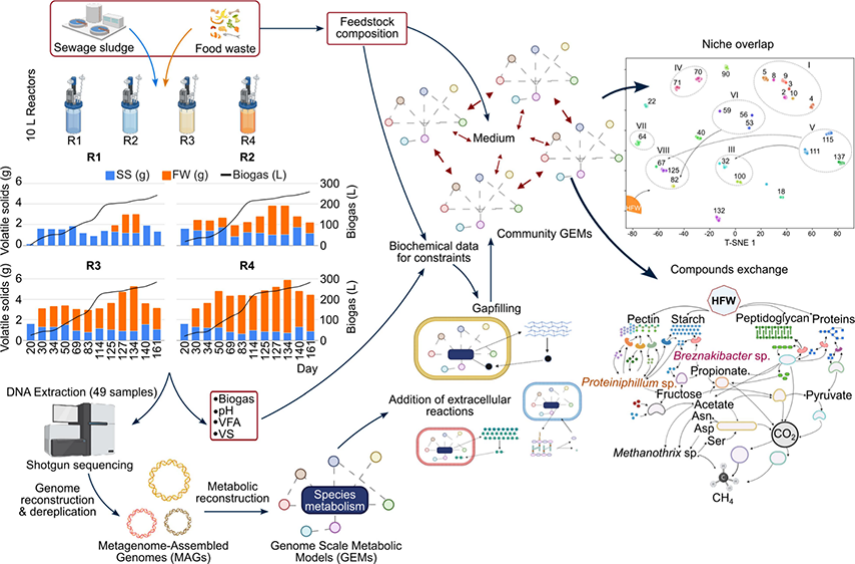

The increasing urbanization has led to rising waste and energy demands, necessitating innovative solutions. A sustainable food waste management approach involves anaerobic codigestion with sewage sludge, enhancing biogas production while managing waste. Although this technology has been successfully tested, the biological mechanisms determining its efficiency are still poorly understood. This study leverages genome-scale metabolic modeling of 138 metagenome-assembled genomes to explore species interactions in lab-scale anaerobic reactors fed with sewage sludge to increasing proportions of food waste. The models showed positive correlations with experimental biogas production (CH_4_: *r* = 0.54, CO_2_: *r* = 0.66), validating their reliability. The dominant methanogen, *Methanothrix* sp., adapted its metabolism based on feedstock, affecting methane yields, which ranged from 2.5 to 3 mmol/g of volatile solids·h with sewage sludge to 10–14 mmol/g of VS·h with high food waste. The integration of extracellular enzymes into the models highlighted the role in methane production of pectin degradation, protein hydrolysis, and lipid metabolism, mediated by *Proteiniphilum* sp., Kiritimatiellae sp., and Olb16 sp. The study identified 475 mutualistic interactions involving amino acid, hydrogen, acetate, and phosphate exchange and 44 competitive interactions in hydrolytic and fermentative processes. These insights can help optimize anaerobic digestion and sustainable waste management in urban settings.

## Abstract 2

Carbon flux exchange prediction in microbial communities is challenging. Findings in this study contribute to fundamental knowledge in microbial ecology and offer practical insights for optimizing anaerobic digestion, especially concerning the simultaneous utilization of diverse feedstocks, including food waste.

## Introduction

The continuous growth of the global population has led to increased urbanization, presenting two major challenges for society: rising energy demand and the surge in urban waste production.^1−3^ The predominant reliance on nonrenewable resources to meet energy needs significantly contributes to the rise in greenhouse gases (GHG) in the atmosphere, notably carbon dioxide (CO_2_) and methane (CH_4_) contributing to the global temperature increase.^4,5^ Globally, approximately 65% of energy is sourced from nonrenewable resources, with higher dependence observed in developing countries like Argentina.^6−8^ Simultaneously, the surge in municipal waste production due to population growth poses a significant environmental challenge.^9^ The activated sludge process, commonly used for municipal wastewater treatment, is effective but incurs a significant energy cost.^10,11^

To address the energy demands of the activated sludge process, biogas generated from the anaerobic digestion (AD) of sewage sludge (SS) emerges as a compelling solution. However, the quantity of biogas produced from primary and secondary sludge digestion often falls short of meeting the full energy requirements of the treatment process.^12^ This shortfall can be attributed, in part, to the limited conversion efficiency resulting from incomplete hydrolysis of organic matter in the systems, primarily composed of microbial cells from aeration tanks. In light of these challenges, addressing increasing energy demands remains a pivotal concern, with waste-to-energy strategies offering a promising avenue to simultaneously mitigate them.^13−15^ Therefore, a more sustainable solution is to codigest the SS with food waste (FW), providing a substrate with higher biodegradable capacity for anaerobic codigestion and improving the efficiency of organic matter conversion to CH_4_.^16,17^ However, codigestion introduces complexity and uncertainty, as different mixing ratios and operating conditions can significantly impact the interactions and dynamics of the microbial community. Understanding how the microbiota responds to various codigestion scenarios and their subsequent effects on process performance and stability is paramount. The AD process exhibits versatility in its utilization of a diverse range of substrates for CH_4_ production, including carbohydrates, lipids, and proteins. Hemicellulose, for instance, can undergo conversion into arabinose and xylose, subsequently metabolizing into glyceraldehyde-3-phosphate via the pentose phosphate pathway.^18^ Additionally, carbohydrates can be degraded into various monomers, such as galactose, mannose, or glucose, which then enter the Embden–Meyerhof pathway, ultimately yielding pyruvate, a pivotal metabolite within AD. Lipids, on the other hand, are degraded by lipases into glycerol and long-chain fatty acids, which readily participate in the β-oxidation pathway. Proteins undergo degradation via proteases, initiating the Stickland pathway, which intersects with β-oxidation through the generation of acyl-CoA^19^. This intricate network of substrate utilization pathways underscores the metabolic flexibility and efficiency of AD systems. Metagenomics and metatranscriptomics, although commonly used, have limitations in unraveling the full complexity of these microbial interactions, as well as strain-resolved dynamics.^20,21^ While reconstructing metagenome-assembled genomes (MAGs) provides insight into organism capabilities and analyzing gene expression offers broader perspectives on adaptive responses, these techniques fall short in capturing the intricate metabolite exchange and species-specific interactions. Metagenomics alone lacks the capacity to fully elucidate these dynamics, and despite the emergence of metabolomics as a valuable tool for quantifying metabolites,^22,23^ applying it to the entire AD process faces significant challenges due to the extensive range of metabolites involved and incomplete pathway understanding. Therefore, these limitations underscore the need for caution in relying solely on them to understand the complexities of AD microbial communities.

To address this need, flux balance analysis (FBA) has emerged as a powerful mathematical tool for studying metabolic processes within microbial communities. This approach relies on the reconstruction of genome-scale metabolic models (GSMMs),^24^ which describe gene-protein-reaction associations for the entire metabolic repertoire of an organism. The models represent the metabolic network in the form of a stoichiometric matrix.^25^ FBA then defines a flux for each reaction by optimizing the linear problem encoded into the matrix for a biological objective function. One commonly used objective is the biomass reaction, reflecting the metabolites consumed for cell generation.^26^ Another possibility is the production of a metabolite of interest, reflecting the optimization of industrial processes. Moreover, FBA assumes that the cells are in a steady state where there is no net accumulation of intermediate metabolites.^27^ The versatility of FBA has been demonstrated across diverse applications, including identifying interactions between tissues (e.g., adipocytes, hepatocytes, and myocytes) in human physiology,^28^ and between microbes, such as *Lactobacillus plantarum* and other species in the gut microbiome or probiotics.^29,30^ Beyond the human organism, it has also been applied to model trophic dependencies in rhizosphere microbial communities^31,32^ and cross-feeding between archaea and bacteria in anaerobic systems.^33,34^ These studies showcase FBA’s potential in predicting microbial interactions, nutrient exchanges, and ecosystem stability, even in highly complex communities.^35,36^ Despite its widespread application, population analysis in systems fed by SS and FW represents a novel extension of FBA’s capabilities.

In this study, the main objective was to improve the understanding of microbial community interactions in anaerobic codigestion through detailed computational models of metabolism integrating information from the feedstocks and explicitly accounting for extracellular enzyme activity. An extension of FBA for microbial communities, cooperative trade-off FBA (ctFBA),^27^ was employed to investigate the metabolic dynamics within communities present in lab-scale anaerobic reactors fed with SS and different proportions of FW. This approach addresses a fundamental issue when modeling complex communities, as the AD microbiome. It balances the community growth rate and microbe cooperation to predict realistic growth of individual species while limiting the computational requirements for large communities.^27^ All findings and results are derived from computational simulations based on metabolic models. Metagenomic data serve as the foundation for constructing metabolic models of the dominant microbial species. Flux simulations encompass diverse codigestion scenarios, featuring varying mixing feedstock ratios. The outcomes offer valuable insights into growth rates, metabolite exchanges, and microbial interactions under different feeding conditions. Furthermore, the predictions are validated against experimental data, which includes total biogas production, CH_4_ yield, volatile fatty acid (VFA) concentration, pH levels, and microbial diversity. This research endeavor aims to uncover optimal codigestion conditions and mechanisms that can enhance bioenergy recovery from organic waste streams, further contributing to collective efforts to address energy demands and environmental challenges in a sustainable manner.

## Materials and Methods

### Metagenome Assembly, Binning, Taxonomy, and Functional Reconstruction

The metagenomic DNA reads used in this study were downloaded from NCBI-SRA (accession number PRJNA544497). The experimental setup and monitored parameters were described previously.^37^ Briefly, four laboratory-scale anaerobic reactors with a working volume of 5 L were operated in semicontinuous mode for 161 days at 35 °C. The inoculum used was collected from the anaerobic digester of a municipal treatment plant located in the Province of Buenos Aires, Argentina, and operated at a mesophilic temperature with a hydraulic retention time (HRT) of 21 days. The digesters received daily feedings and were intermittently stirred with a cycle of 30 min on and 60 min off. Feeding began with a gradual mix of primary and waste activated sludge from the same WWTP until an organic loading rate (OLR) of 1.5 g volatile solids (VS) L^–1^ day^–1^ was reached. Reactors 2 to 4 were then gradually adjusted to OLRs of 2.5, 3.5, and 4.5 g L^–1^ day^–1^ with FW additions, respectively. On day 125, all digesters, including the control, received an extra 2 g VS L^–1^ day^–1^ of FW for 2 weeks, before returning to the initial feeding conditions. Twelve sampling points were selected at approximately regular intervals over the 161 days of digester operation (days 8, 20, 30, 34, 69, 83, 114, 125, 128, 135, 140, and 161). To facilitate the interpretation, samples were categorized into three groups according to the quantity of FW per liter of VS: low FW (LFW) for those with less than 0.2 g FW/L VS, medium FW (MFW) for samples ranging from 0.2 to 0.6 g FW/L VS, and high FW (HFW) for samples exceeding 0.6 g FW/L VS (Table S1). The total and volatile solids (TS and VS) of the inoculum as well as other relevant parameters are detailed in the Supporting Information.

In order to improve the assembly and genome reconstruction, raw reads were quality-filtered and trimmed, removing fragments shorter than 100 bp and with quality <30 (Q30) using Trimmomatic software (v. 0.39). The first step for the study of metabolite fluxes in the community was the reconstruction of MAGs. GSMM reconstruction, in contrast to the functional study at the population level, requires good-quality genomes with a high percentage of completeness and a low percentage of contamination. In order to fulfill these criteria, a cutoff of 90% of completeness and 5% of contamination was set. A combination of different metagenome assembly and binning approaches was performed to obtain an exhaustive number of MAGs with a completeness of at least 90%. A first assembly using all samples together (*n* = 49) with two different software was used: MEGAHIT (v. 1.2.9) and metaSPADES (v. 3.15.5). A second approach on the metagenome assembly was performed by using exclusively the 36 samples containing FW. Furthermore, in order to increase the quality of the most abundant MAGs, a third assembly was conducted focusing the efforts on a subset of 6 specific samples obtained from reactor 3 (days 50, 69, 114, 128, and 135). The contigs were grouped by means of different software: MetaBAT (v. 1.2.15) using “sensitive” parameters and maxBin (v. 2.2.7) and vamB (v. 3.0.9) with default parameters.^38,39^ The quality of each MAG was calculated by checkM (v. 1.0.2), and only those with a completeness greater than 50% were retained. Using dREP software (v. 3.4.0),^40^ genome–genome comparisons were performed for the entire collection of MAGs to remove redundancy. Finally, to verify whether MAGs of higher quality corresponding to the species retrieved in this study have been identified before, a second dereplication was performed against the AD database.^41^

Taxonomic classification of the MAGs was carried out by GTDB-Tk (v. 1.7.015) by default parameters, and the open reading frames (ORFs) were detected with Prodigal (v. 2.6.3) using the respective domain as the only parameter. The cellular localization of the predicted enzymes was detected by a combination of two software: the putative signal peptides were detected through the standalone version of DeepSig,^42^ and the putative extracellular proteins were confirmed by BUSCA.^43^ Once the extracellular enzymes were identified, a manual curation of the functional annotation was performed by combining two different approaches: first, Ghost-KOALA automatic annotation server using the KEGG database was used,^44^ and second, eggNOG (v. 5.0) was implemented using eggNOG-mapper (v. 2.0.1)^45^ with default parameters and DIAMOND software to align the sequences.

### Genome-Scale Metabolic Model Reconstruction and Simulation

Biochemical results from the inoculum and the first days of operation were used as starters for calculating the medium composition of the SS fraction. A special focus on similar studies was considered to establish the concentration of relevant compounds in this feedstock, such as phosphate, ammonia, and vitamins.^46−49^ The determination of FW composition was possible thanks to the Virtual Metabolic Human (VMH) database (https://www.vmh.life/) by searching each substrate (apple, banana, bread, cabbage, chicken, eggplant, mince, and onion) and transforming the data into total mmol of each food in each sample (Tables S2 and S3). By knowing the total grams of SS and FW per VS added in the reactor, a final concentration of mmol gVS^–1^ h^–1^ was obtained (Table S4).

The draft GSMMs of microbial species with a high-quality MAG (*n* = 138, Table S5 and Supplementary Data S1) underwent reconstruction and gap-filling using gapseq (v. 1.2),^50^ predicting transporters and biochemical reactions to ensure viability in anoxic conditions. Specific parameters were set up in each step of the reconstruction of the models: −*p* all and −*b* 200 in the gapseq find step; −*b* 200 in the gapseq find-transport step; −*u* 200 and −*l* 50 in the gapseq draft step. Prior to the gap-filling step, the extracellular enzyme functions were correlated to the gapseq database to determine the reactions involved. A set of extracellular reactions were manually added to the “draft” models in the ante-last step of the gapseq pipeline (Supplementary Data S2). These added reactions are described in Table S6. Functional characterization of the models was corroborated with KEMET.^51^ Then, the gap-filling of each metabolic model was performed by using the predicted medium except for the case of the archaeal models, where either an acetate or H_2_–CO_2_-enriched medium (provided by gapseq) was used according to the genera assigned. The illustrated experimental design is provided in Figure S1.

All metabolic models obtained by gapseq were analyzed by COBRAby^49^ and MICOM (v. 0.32.4),^27^ which allows estimating microbial growth rates and establishing interactions between the different organisms through the cooperative trade-off flux balance analysis (ctFBA) approach (Supplementary Data S3). This also calculates the import and export fluxes of each MAG–compound pair and checks whether the products of one MAG can be used as substrates by another. To contrast growth rate predictions with an independent measure of microbial growth, the peak-to-trough ratio (PTR) was calculated using CoPTR.^52^ Cooperative trade-off FBA was performed by using microbial relative abundance in each sample as input and by setting as “True” the pfba parameter in MICOM to obtain fluxes by parsimonious FBA. Together with the constraints given by the media and VFA concentration measurements, this ensured that the space of the possible solutions was substantially limited. Moreover, several exchange reactions in individual GSMMs were checked manually based on the literature on isolated organisms (Table S7). In order to establish the optimal trade-off parameter (ct) value, an initial exploration through 0.4 to 1 was done and the production of CH_4_ and CO_2_ in combination with the growth rate of the archaeal community determined that the best ct was 0.7 similarly to other anaerobic systems^53^ (for more details, see the “Community flux balance analysis” section in the Supporting Information). MAGs, the script for adding extracellular reactions, files and scripts for community simulations, and FBA results (including fluxes for each species, community exchanges with the medium, and intracellular reactions) are all available in Supplementary Data (S1–S6) on Zenodo (https://doi.org/10.5281/zenodo.14704579).

A more detailed methodology is described in the Supporting Information.

### Microbial Interaction Network Reconstruction

To elucidate the dynamics of interactions among diverse organisms within the studied community, a multistage approach was employed. First, Pearson correlation analysis using MAGs’ abundance across all the samples was performed to identify potential ecological relationships. A stringent filter was applied, excluding absolute correlations lower than 0.65 (*p*-value <0.001), to ensure robust identification of significant co-occurrences. Second, negative correlations between MAG abundances from distinct organisms were interpreted as potential indications of competition, particularly when species shared similar preferences for resources. Overlap between the ecological niches were defined by applying a threshold on the import flux. Species importing the same compounds at a rate greater than 0.01 mmol DW^–1^ h^–1^ were considered to potentially establish competitive dynamics within the same ecological niche. Third, positive correlations were analyzed to identify mutualistic interactions, defined by metabolic exchanges where the metabolic products of one organism (export: >0.01 mmol DW^–1^ h^–1^) served as substrates for another (import: ←0.01 mmol DW^–1^ h^–1^). These stages collectively provided a systematic framework for distinguishing between competitive and cooperative interactions within the microbial community.

To visualize the ecological niches, t-distributed stochastic neighbor embedding (t-SNE) was applied to condense the import flux data into two dimensions. Niches were then delineated by manually grouping clusters of spatially proximate species on the resulting map, reflecting similarities in metabolite consumption patterns.

## Results and Discussion

### Reactor Performance, Feedstock, and Microbiome Characterization

Microbial samples from four laboratory-scale anaerobic digesters were used to reconstruct the community inhabitant genomes and, subsequently, the GSMMs. Details regarding the reactor’s performance were previously discussed.^54^ Briefly, each digester was inoculated with anaerobic sludge from a wastewater treatment plant and initially fed with a combination of primary and secondary sludge. Subsequently, each reactor, except for the control (reactor 1), received FW in addition to the SS, increasing the VS loading at a 10% daily rate until the predefined targets for organic loading rates of 2.5, 3.5, and 4.5 g VS L^–1^ per day were achieved in reactors 2, 3, and 4, respectively (Figure S2). Once the feed reached the desired organic loading, the feeding regimen was maintained for 5 hydraulic retention times (HRT). Biogas production exhibited a significant increase in the FW-supplemented reactors compared with the control. However, while the total CH_4_ production increased with a higher proportion of FW, the specific CH_4_ yield (L CH_4_/g VS) decreased (Table S1). The composition assessment of FW revealed a significant presence of various carbohydrates: starch, fructose, glucose, sucrose, and maltose. Additionally, a high proportion of glutamic acid was identified, primarily originating from chicken and minced meat, as previously observed in other food wastes.^55−59^ The estimates of the FW composition accurately reflect the concentrations of carbohydrates, lipids, and proteins. Biochemical analyses revealed that 74% of the biomolecules (i.e., carbohydrates, proteins, lipids, nucleotides, and vitamins) consisted of carbohydrates. Additionally, estimates derived from the VMH database, which comprehensively catalogues human and gut microbial metabolism and correlates these data with nutritional information, indicated a slightly lower representation of 72.3%. Finally, for the protein and lipid proportion, findings indicated 9 and 18% compositions, respectively (Table S2).

A tailored metagenome assembly and scaffold clustering were performed to obtain 104 high-quality (HQ) MAGs. Additionally, all MAGs were dereplicated together with the AD database composed of nearly 10,000 MAGs reconstructed from metagenomes of anaerobic studies.^60^ The final collection includes 138 HQ MAGs representing 55% of the total sequenced reads and 77% of the metagenome-assembled contigs. The microbial community represented by the HQ MAGs included 27 different phyla (Figure S3), with the highest number of representatives being Bacteroidota (*n* = 27), Firmicutes_A (*n* = 18), Desulfobacterota (*n* = 12), Actinobacteriota (*n* = 10), and Halobacteriota (*n* = 9). Interestingly, 9 of them represent 50% of the total average abundance of the MAGs in all samples. The most dominant species belongs to the phylum Chloroflexota, Anaerolineaceae sp. 076, with an average relative abundance of 8 ± 5%, while the next 4 dominant MAGs are all from the order Bacteroidales (with average relative abundance between 5 and 7%). Although archaeal species represented less than 10% of the total community, *Methanothrix* sp. ranked seventh with 3.7% of the relative abundance (Figure S4). This genus was formerly known as *Methanosaeta*.^61^

### Correlation between Predicted and Measured Biogas Production

**Figure 1:**
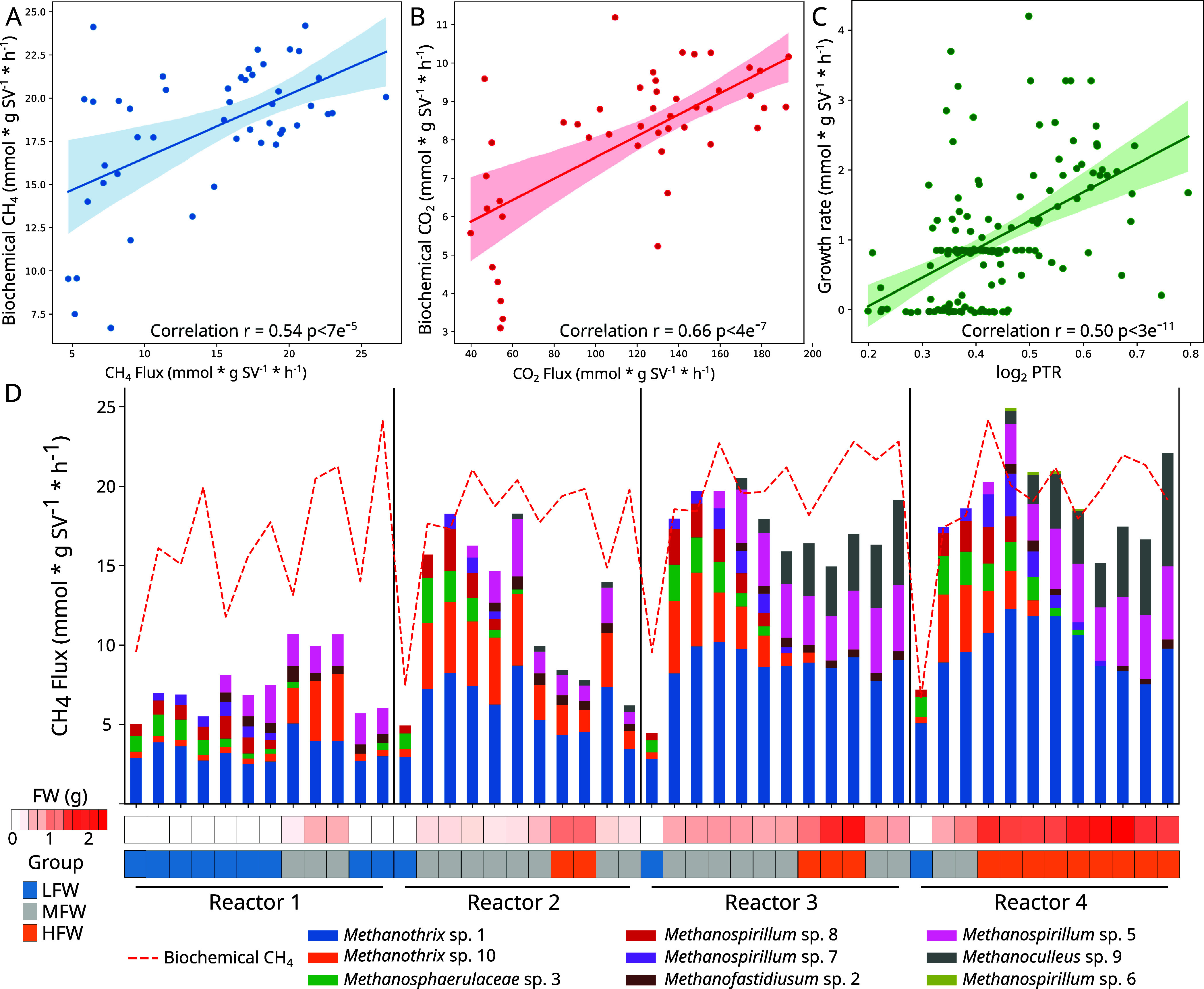
Validation of GSMMs with model-independent measurements. Correlation between fluxes obtained from the simulations (*x*-axis) and biochemical measurements (*y*-axis) of CH_4_ (A) and CO_2_ (B) production rates. Each data point corresponds to one of the 49 samples, and the simulation results represent the net uptake/secretion flux of the community. Samples were categorized into three groups based on the amount of FW provided to the reactor: low (LFW), medium (MFW), and high (HFW). Correlations between the archaeal growth rate obtained from the simulations (*y*-axis) and those predicted from metagenomics (*x*-axis, log_2_ PTR) are shown in panel C. Each data point represents the growth rate of a specific archaeon in a given sample. The predicted methane production rate for each archaeal species over time is displayed in panel D, with bars representing the total predicted CH_4_ flux and the dashed line indicating the corresponding experimental value. On the bottom, a heat map illustrates the amount of FW supplied at every time point, while a second heat map represents the FW group classification.

Microbial community simulations employed a steady-state approach, known as ctFBA, which balances interspecies cooperation to investigate the pathways and interactions involved in substrate degradation and CH_4_ production. The aim was to reproduce *in silico* the most likely metabolic equilibrium established within each community while ensuring alignment with experimental data. The simulations were conducted using community-level GSMMs tailored to the microbial compositions of each of the 49 metagenomic samples (Table S1). For each sample, species with a relative abundance greater than 0.1% were included in the simulations by reconstructing and integrating all the corresponding species-level GSMMs. Additionally, feedstock composition and VFA concentrations were applied as model constraints, while CH_4_ and CO_2_ production fluxes were used to evaluate the model performance. The success of this cooperative strategy was assessed by maximizing the correlation between the predicted CH_4_ flux and observed production through the trade-off parameter (Materials and Methods), thus ensuring that each species achieves maximum growth within the collaborative solution space. Correlations between the measured biochemical parameters (i.e., CO_2_ and CH_4_) relative to grams of VS and the flux resulting from the modeling (CH_4_: *r* = 0.54, *p*-value <7 × 10^–5^, Figure 1A; CO_2_: *r* = 0.66, *p*-value <4 × 10^–7^, Figure 1B and Supplementary Data S4) supported the reliability of the simulations. Moreover, the archaeal growth rate from the simulations was compared with the replication rate estimation obtained using the DNA reads (log_2_ PTR), resulting in a positive correlation (*r* = 0.50, *p*-value <3 × 10^–11^), which again indicated good agreement between model predictions and independent estimates (Figure 1C).

Subsequently, a detailed analysis of the predominant *Methanothrix* sp. 1 was undertaken. Despite acetate being the main energy substrate of this organism from isolation studies,^62^ it has been observed that it can also utilize CO_2_ by reducing it through direct electron transfer in the reductive hexulose-phosphate (RHP) pathway.^63^ According to the flux balance simulation, this species consumes a higher amount of acetate in the presence of FW compared to that when SS is used as a sole substrate. When comparing the total CH_4_ production, the simulations predicted lower yields than those observed experimentally. This discrepancy may be attributed to the more precise characterization and quantification of feedstock in samples with a higher food waste (FW) content. In these samples, the exact amounts of each component were measured before the reactor. In contrast, the SS samples received heterogeneous feedstock on a weekly basis. Integrating additional constraints from other -omics layers could enhance the simulation results, leading to fluxes that more accurately reflect the experimental data.^53^

Surprisingly, samples with a higher organic load demonstrated an increased CO_2_ uptake rate. This phenomenon may be attributed to higher fermentative activity in response to the influx of new substrates, mainly polymers, triggering a cascade in the hydrolytic and fermentative steps of AD, leading to an increase in VFA availability. The rise in the relative abundance of syntrophic species from Synergistaceae sp. 40 and Treponematales sp. 132 is considered a contributing factor to this organic load-induced cascade, resulting in enhanced CO_2_ production and subsequent consumption by the methanogenic guild. Furthermore, FBA underscored the significance of less abundant hydrogenotrophic archaea (i.e., *Methanospirillum* sp. 5 and *Methanoculleus* sp. 9), whose pivotal role in CH_4_ production became evident, particularly in samples with higher concentrations of FW (Figure 1D and Supplementary Data S5).

### Metabolite Exchanges Are Associated with the Codigestion Balance

**Figure 2:**
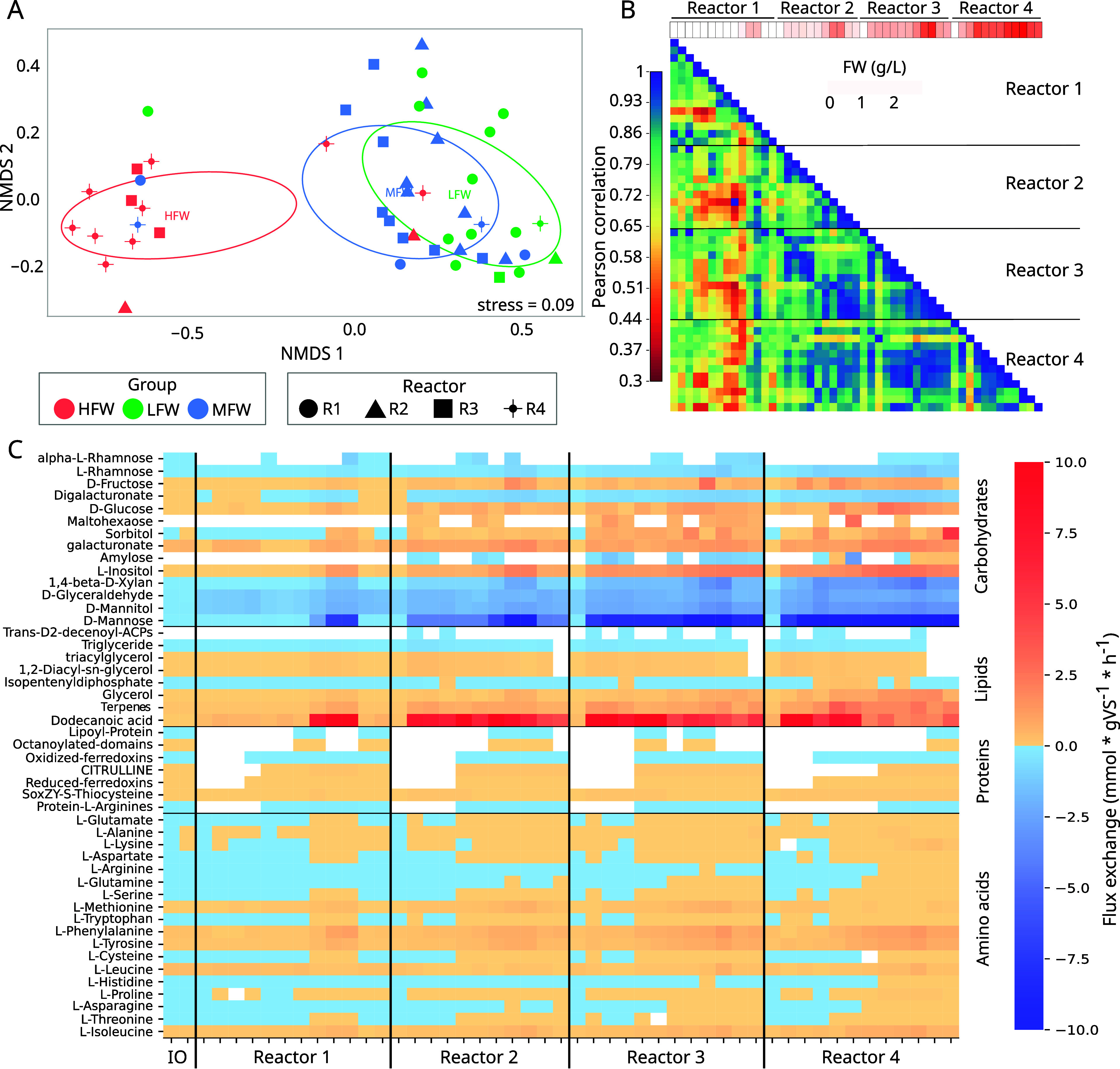
Total sum of uptake/secretion fluxes of the microbiota. NMDS of all exchange metabolites between the medium and the community in each sample (A). Similarity matrix among each of the samples using a flux exchange matrix between the medium and the microbial consortia (B). Flux exchange of the different pectin derivatives, lipids, proteins, and amino acids between the medium and the community in each sample (C). Negative values represent consumption of the compound, and positive values indicate secretion to the medium.

In order to visualize the distribution of the populations in each sample, a nonmetric multidimensional scaling was built using the exchange fluxes (total sum of microbial uptake/secretion) (NMDS, Figure 2A and Supplementary Data S4). This revealed the formation of sample clusters based on the FW content (LFW, MFW, and HFW), indicating that the simulated microbial dynamics effectively captured the diverse environmental conditions arising from increasing FW supplementation. Applying the same statistical analysis to the MAG abundance data revealed consistent clustering patterns, underscoring a strong correlation between the simulated microbial dynamics and the observed community structure (Figure S5). The gradual shift in the microbial populations, triggered by the introduction of a new substrate, manifested a pronounced separation between the HFW and reactors with lower FW concentration. When comparing exchange fluxes between samples, lower correlations were observed comparing reactor 1 and the others (Figure 2B). The increase in FW led to a transformative impact on the community, influencing the consumption and production of various compounds, e.g., sucrose, mannose, and rhamnose. Notably, reactors with a higher FW proportion (i.e., R3 and R4) demonstrated a higher stability, as measured by dissimilarity between exchange fluxes within the same reactor. Although all reactors operated on heterogeneous substrates, the higher FW proportion in reactors R3 and R4 contributed to this increased stability. This result aligns with the observed enhanced stability in microbiota in the presence of heterogeneous substrates.^64^

Examining individual exchanges is significant to highlight the three principal groups of carbon-based compounds: carbohydrates, lipids, and proteins (amino acids, AA) (Figure 2C and Figure S7). Complex carbohydrates showed net consumption in all cases except for amylose in the high FW concentration samples. Surprisingly, glucose did not show a net consumption, but sucrose and maltose were utilized in most of the samples. Possibly, the transport efficiency or metabolic preferences for these disaccharides are high, and the energy needed for their direct import is lower than the energy required to export hydrolytic enzymes and then import the monosaccharides. Despite the uniform intake of these disaccharides over time in the four reactors, the primary organisms utilizing them were replaced by species of Bacteroidales. In HFW samples, the exchange fluxes of disaccharides were increased on Treponematales spp. and *Propionimicrobium* spp., showing major incorporation when compared with LFW (Supplementary Data S5).

While most of the predicted lipid exchange fluxes were low, specific compounds stood out, such as dodecanoic acid (ddca), terpenes, and glycerol. These are directly derived from the estimates of compounds within the FW and are predicted to be accumulated. However, key species play a role in their degradation, Bacteria sp. 134, *Syntrophomonas* sp. 66, and Syntrophomonadaceae sp. 68; specifically, the increased concentration of ddca in the medium correlates with a higher abundance of *Syntrophales* sp. 92, which concomitantly produces acetate through the beta-oxidation pathway.

Additionally, auxotrophy in AA biosynthesis is known and exchanges are pivotal for maintaining high microbiome diversity and long-term stability.^65^ Indeed, AA plays a critical role in various metabolic pathways, serving as central components in the production of pyruvate through transamination and deamination.^66^ Despite their scarcity in the feedstock (Table S3), the exchanges predicted within the community and with the medium were quite low. Interestingly, in the *in silico* predictions, none of the AA was completely consumed in the HFW samples. This is likely due to the rapid release of hydrolytic enzymes. These findings support previous evidence that serine, methionine, and aspartate are not fully utilized in mixed reactors.^67,68^l-Lysine and l-proline demonstrate a dual behavior of consumption and accumulation. Negative values in samples containing only SS indicate net consumption, whereas accumulation occurs in all other samples. The primary organism responsible for AA production, *Ancrocorticia* sp. 70, can synthesize l-lysine from aspartate or l-homoserine, with a higher flux observed in the case of l-aspartate. *Methanothrix* sp. 1 showed higher levels of consumption of l-alanine, l-aspartate, l-asparagine, and l-threonine than other archaea (Figure S8 and Table S8), which is possibly correlated with the highest CH_4_ production (Spearman correlation coefficient *r* = 0.45 and *r* = 0.55, respectively, *p*-value <1 × 10^–3^). A modest Spearman correlation was also found between the archaeon abundance and the FW (*r* = 0.45, *p*-value <1 × 10^–3^); however, the correlation significantly increased to 0.80 when considering the activity of 12 out of the 14 reactions where l-aspartate participates. This difference suggests that activities related to some compounds, such as l-aspartate, provide a more nuanced understanding of AA association with the varying feedstock conditions. Finally, l-cysteine can cause an increase in CH_4_ production through the acetoclastic pathway by activating the conversion of glucose to acetic acid.^69^ However, the addition of the cosubstrate resulted in the accumulation of l-cysteine, indicating that its contribution to CH_4_ production could not be as important as expected. These discrepancies underscore the necessity for more precise feedstock characterization, incorporation of additional environmental variables such as metabolite concentration (e.g., AA) and temperature fluctuations, and refinement of the metabolic model to account for microbial interactions and substrate heterogeneity, thereby enhancing the accuracy of predictions.

### Extracellular Reaction Dynamics Reflects the Concentration of Cosubstrates

Given the pivotal role of extracellular enzymes in the degradation of complex substrates such as FW, associated reactions were manually included in the models to explicitly capture their activity in the codigestion process (Table S6). Subcellular localization of enzymes was assigned for each MAG, and the correct compartment was associated with catabolic transformations. A total of 59 MAGs presented extracellular enzymes, mostly Bacteroidota (*n* = 19) and Firmicutes (*n* = 10), aligning with their common association with hydrolytic functions in anaerobic environments.^70^ Cooperative trade-off FBA highlighted a positive Pearson correlation between FW concentration and the total flux through extracellular reactions (*r* = 0.85, *p*-value <1 × 10^–5^, Table S9), demonstrating a stronger association compared to intracellular reactions (*r* = 0.55, *p*-value <1 × 10^–14^, Supplementary Data S6). These findings quantitatively confirm the key role of extracellular enzymes in FW degradation.

**Figure 3:**
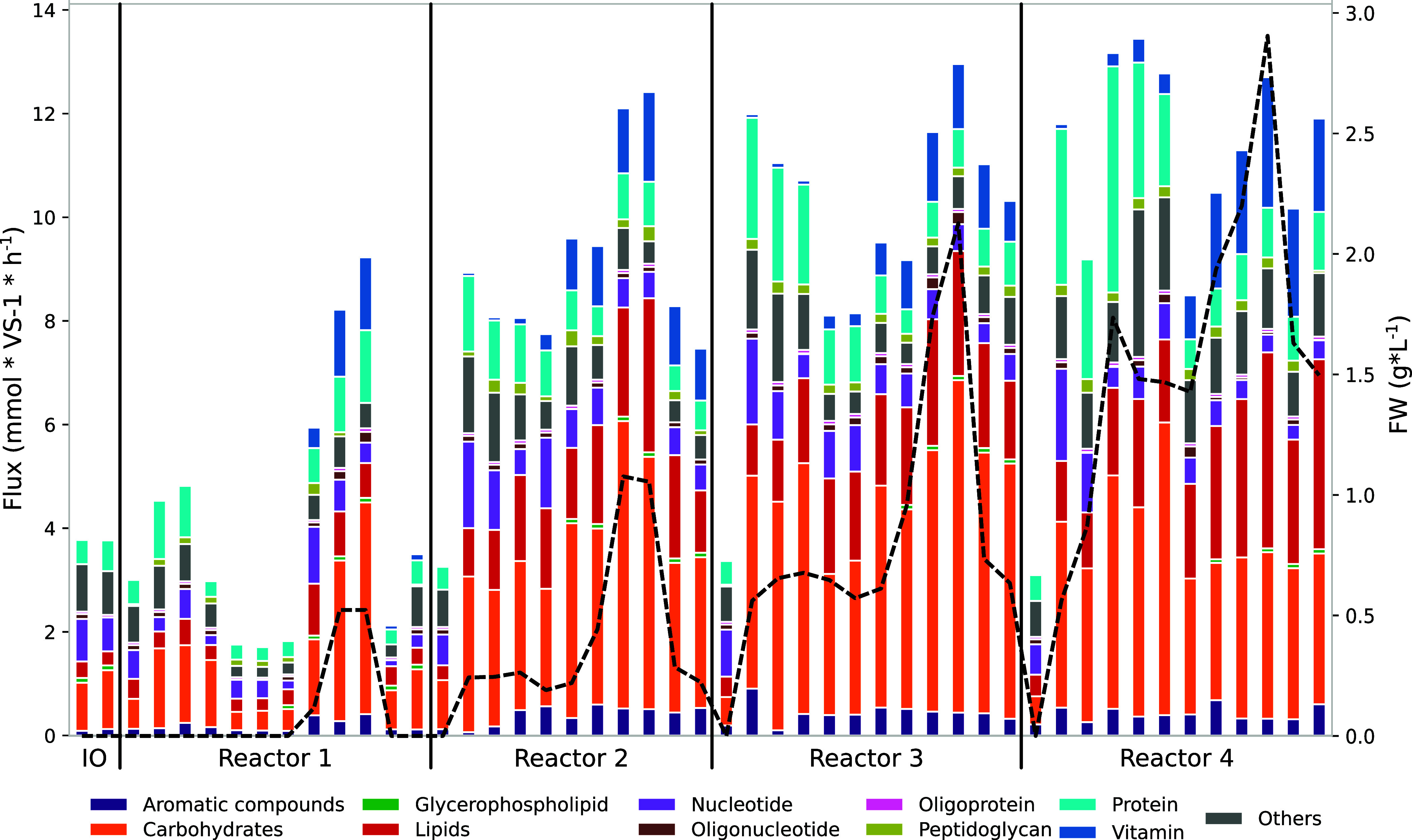
Extracellular activity. Predicted extracellular reaction fluxes in mmol VS^–1^ h^–1^ grouped by type of molecule involved in the reaction. The black dashed line represents the concentration in grams per liter of FW measured in the reactor.

The primary components making up the FW are carbohydrates, constituting 12.57% of the dry weight (Table S2). This composition finds a correspondence in the overall extracellular activity, with 34 ± 8% attributed to extracellular enzymes involved in carbohydrate degradation (Figure 3). In particular, the reaction with the largest flux was exopolygalacturonase (KEGG: R04320, EC3.2.1.67), involved in pectin degradation and production of d-galacturonate from pectate. This reaction has been reported in several organisms in different anaerobic environments,^71−73^ and in our case, it was exploited by a single MAG, *Proteiniphilum* sp. 26. While the presence of other pectin-hydrolytic enzymes was verified in multiple organisms, it was found that two taxa in particular, Prolixibacteraceae sp. 107 and *Bacteroides* sp. 25, harbor extracellular enzymes that can potentially digest pectin (Tables S9 and S10). However, the former exhibits a low flux, while *Bacteroides* sp. 25 showed sustained flux values for the poly(1,4-alpha-d-galacturonate) glycanohydrolase reaction, which removes a digalacturonate unit from the pectate chain. The abundance of this MAG increased with the addition of FW and decreased at a high FW content, possibly displaced by *Proteiniphilum* sp. 26 due to competition for the same substrate. Thus, the GSMMs confirm what had been hypothesized by inspecting the metabolic capabilities of the organisms and show that a major fraction of carbohydrate degradation can be explained by the activity of a single taxon at each time point. This observation underscores the significance of pectin degradation as a crucial step in the process with low functional redundancy.

While the protein content in FW is two times higher than that of lipids, fluxes associated with each compound type were similar, representing 13 ± 6% for proteins and 16 ± 6% for lipids. This suggests that protein fluxes may be predominantly intracellular, which could account for their lower extracellular fluxes relative to the high protein content in the FW. Thirty species were predicted as active in protein degradation in at least one sample. Anaerolineaceae sp. 76 encoded 91 proteases, but its abundance was higher in samples with a low FW content, suggesting its relevance in SS.^74^ Two MAGs had a positive abundance correlation with the influx of FW and were the second and third community members with the highest protease fluxes: Syntrophomonadaceae sp. 64 (*n* = 76 proteolytic enzymes) and *Ancrocorticia* sp. 70 (*n* = 55). While lipid degradation enzymes were restricted to only 21 MAGs, one had a significant number of lipase fluxes, reaching a count of 116 by *Gordonia* sp. 69. This genus has been frequently associated with foaming issues in AD of sludge.^75^ It may have been introduced in the inoculum and persisted throughout the experiment, but when FW was supplemented, it was displaced by other taxa. One of them could be *Ancrocorticia* sp. 70, as in the models, it contributed to lipid degradation similarly to *Gordonia* sp. 69.

### Interactions and Competitive Dynamics within Microbial Populations

Strong negative and positive correlations between various species were selected by applying a Pearson correlation to the MAG abundance across conditions (|*r*| > 0.65 and *p*-value <0.001). This resulted in a complex network of relationships that are likely to influence community dynamics and function. To specifically focus on potential ecological niche competition,^76^ negative correlation pairs showing a preference for the same substrates (resources competition) were included. This adjustment is crucial because determining whether a negative correlation between a species pair is a result of competitive exclusion or niche filtering remains a challenging task. Conversely, positive correlations may suggest mutualism, where both organisms benefit from the interaction. In this case, pairs of interacting organisms were assessed for using each other’s metabolic products as substrates. This approach led to the identification of 475 beneficial interactions (from 544 positive correlations) involving 114 MAGs, while 44 competitive interactions (from 57 negative correlations) were identified among 30 MAGs. The lower occurrence of competitive interactions in this type of microbiota aligns with previous findings in metabolic models and abundance-based correlations.^77,78^

**Figure 4:**
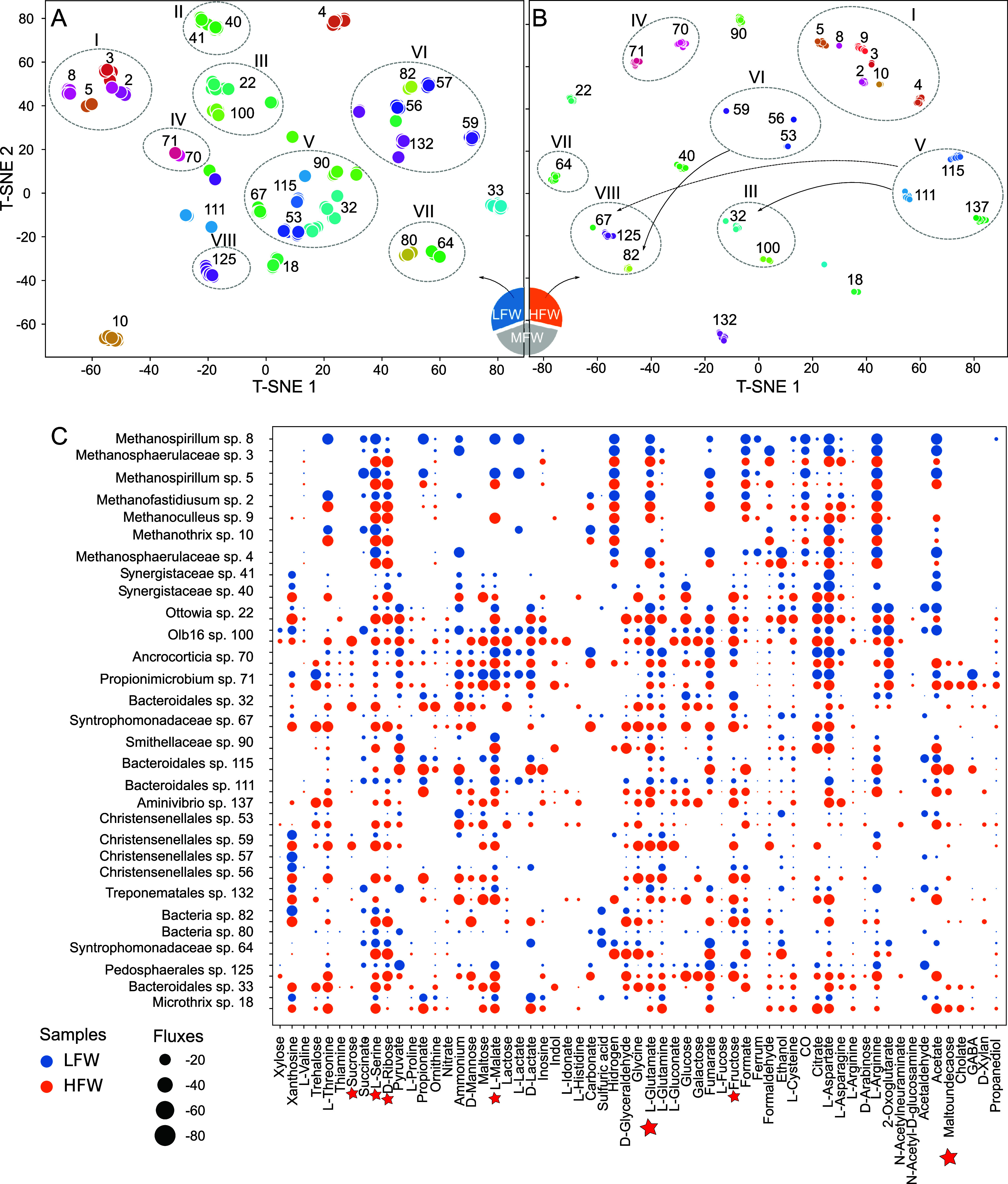
Map of competition based on growth niche. Import fluxes for GSMMs in samples having LFW concentration ((A) lower than 0.2 g L^–1^, 15 samples) and HFW concentration ((B) higher than 0.9 g L^–1^, 14 samples), condensed into two dimensions through t-distributed stochastic neighbor embedding (t-SNE). Each circle on the map represents a species in a specific sample and is surrounded by a Roman numeral circle representing its microbial niche. Niches were manually delineated by grouping clusters of species that were spatially close on the t-SNE map, reflecting similar metabolite consumption patterns. The color corresponds to the species’ identity. Species located close to each other on the map exhibit similar metabolite consumption patterns. Import fluxes of the main compounds for the organisms shown in A and B are summarized in C. Red stars represent the metabolites with a higher correlation with the FW concentration.

Numerous competitive interactions occur at the hydrolytic and fermentative levels involving substances such as starch, xylan, glucose, propionate, and various compounds from the TCA cycle; additionally, different AA groups participate in negative interactions (Table S3). In general, it was observed that resource competition had a greater impact among hydrogenotrophic archaea, occupying the same ecological niche in both feedstock conditions: LFW and HFW (Figure 4). A noteworthy observation is that the hydrogenotrophic archaea Methanosphaerulaceae spp. 3 and 4 (also known as *Methanoregulaceae*)^79^ potentially competed with various Christensenellales spp. for acetate. However, these Clostridia-class bacteria are also reported to engage in a syntrophic interaction by oxidizing acetate and producing CO_2_ and H_2_,^80^ which support the hydrogenotrophic growth of the archaea. Moreover, these archaea and bacteria competed for l-aspartate, l-glutamine, and l-alanine. A potential explanation is that, while syntrophic relationships may occur due to metabolic dependencies in the ecosystem,^81^ varying metabolite concentrations could satisfy both organisms, potentially reducing direct interactions.^82^ However, under conditions where the feedstock consisted solely of SS, a limited variation in the ecological niches occupied by different bacterial species was observed. This may be attributed to the low diversity of resources available (Figure 4A–C). Conversely, in the case of HFW (Figure 4B,C), a different behavior was noted, where bacteria changed in different samples, primarily due to the availability of a new resource and their ability to degrade certain compounds. This allowed them to make substrates available for the rest of the community, avoiding competition among themselves. One of the most extreme cases can be observed as Treponematales sp. 132 occupies the same ecological niche as Bacteria sp. 82 in samples with little or no FW. Meanwhile, this overlap is not observed in samples with a high concentration of FW since they occupy very distant niches.

Furthermore, multiple phyla were observed to engage exclusively in mutualistic interactions (see Materials and Methods subsection Microbial Interaction Network Reconstruction), enhancing the correlation network based on organism abundances, with particular significance attributed to the Firmicutes phylum (*n* = 50), thus corroborating the conclusions drawn in prior investigations.^78^ For example, a similar trend was observed in microbiota fed entirely with food waste.^83^ Moreover, Synergistetes (referred to as Syngergistota in this work) have been speculated to be involved in several mutualistic interphylum interactions, similar to this study^36,84^ (Figure S8A). Interestingly, it was observed that the majority of interactions were evenly distributed across LFW, MFW, and HFW (Figure S8B). Although there were slightly more positive interactions in LFW, the number of compounds involved in interactions between organisms increased by 35% in HFW samples (Figure S8C). All archaea were found to participate in mutualistic interactions, which is expected, as they represent the final step in the AD trophic chain. A higher prevalence of mutualistic interactions between the Bacteroidota and Firmicutes phyla than within each phylum individually was also observed (Figures S2 and S8A). Similar interactions have been detected in previous simulations based on the presence of bacteria detected via ribosomal sequencing in the gut^85^ and thermophilic reactors.^36^ The prevalence of positive interphylum interactions suggests that specialized organisms involved in the digestion of specific compounds are closely related, competing for the same ecological niche, while less closely related species have different metabolisms and can establish positive interactions.^86,87^

**Figure 5:**
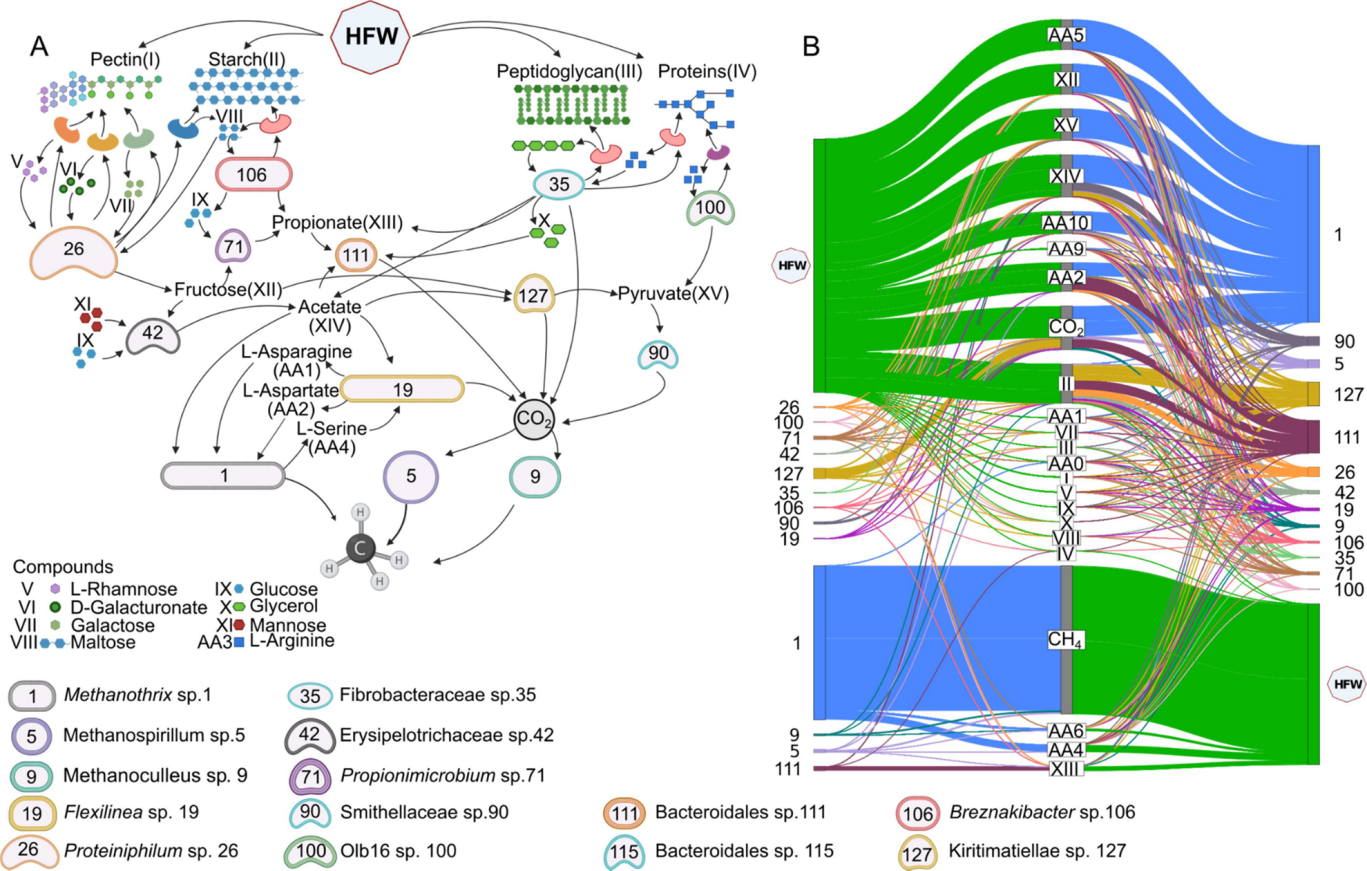
Main interactions in HFW samples. A complex web of interactions unfolds as diverse organisms engage in symbiotic relationships, nutrient cycling, and decomposition within the HFW environments (A). Predicted fluxes between the main organisms are shown in panel A. Right-to-center lines represent secretion of different metabolites (B). Left-to-center lines represent consumption.

Notably, four mutualistic interactions persisted throughout the entire experiment. One of these involved the most abundant archaea, *Methanothrix* sp. 1 and *Flexilinea* sp. 19 (Figure 5). Among the numerous compounds predicted to be exchanged between them, AA exhibited the highest flux alongside hydrogen (H_2_). Specifically, l-asparagine and l-aspartate had the highest flux as substrates for the archaeon, while *Flexilinea* sp. 19 primarily utilized l-serine. This reinforces the significance of AA in bacterial-archaeal methanogenic interactions, as discussed in prior research.^88^ Interestingly, a possible exchange of H_2_ between the fermentative bacteria *Flexilinea* sp. 19 and the acetoclastic *Methanothrix* sp. 1 was predicted. Although the simulations did not include molecules typically associated with direct interspecies electron transfer (DIET), H_2_ can effectively function as an electron donor. This behavior could be a simulation artifact, where H_2_, in the absence of explicit DIET molecules, mimics electron transfer dynamics in a syntrophic relationship. In this context, H_2_ may act as a mediator for electron flow between species, analogous to electron transfer processes observed in a DIET-driven metabolism.

In each mutualistic interaction pair, the most recurrent compounds that are predicted to be shared include acetate, phosphate, l-cysteine, and l-serine. Kiritimatiellae sp. 127 and *Propionimicrobium* sp. 71, despite their proficiency in consuming pectin, are also involved in acetate utilization and propionate production, respectively. *Breznakibacter* sp. 106 appears to be the primary factor responsible for starch degradation in HFW. The hypothesis posits that a fraction of the glucose and maltose, obtained from starch, is liberated into the medium, rendering it available to the microorganisms residing within the microbiota.

The importance of AA exchange among species has been discussed previously, but the central role of acetate in the exchanges of AA is noteworthy. The predominance of mutualistic interactions over competition could be due to the steady supply of SS in all reactors coupled with the incremental addition of FW. The shift in feedstock composition may have driven the microbial community to adapt, particularly affecting the hydrolytic and fermentative stages, which seem to play a crucial role in shaping the trophic dynamics. Spatial occupancy of different species could vary based on the proportion of FW, suggesting that the presence of a secondary feedstock in reactors could alleviate competition between microbial populations.

The primary objective of this research was to elucidate the metabolic interplay within anaerobic codigestion microbial consortia, with the ultimate aim of improving biogas production from varied organic waste streams in real-world applications. In summary, the study generated GSMMs that explain biogas production across a gradient of SS/FW proportions and provide insight into the activity of the associated microbiota. The integration of reactor biochemical data, metagenomic analysis, and metabolic modeling provides a comprehensive view of the community’s structure and function. This work therefore elucidates not only how the abundance or presence of certain organisms is modified due to the modulation of feedstock but also how these changes reflect variations in the exchange of metabolites. These results confirm hypotheses formulated based on the microbial functional potential and propose new ecological roles for major microbial players within the controlled ecosystem. More specifically, the results highlight the importance of extracellular enzymes in degrading FW by means of GSMMs. Our findings indicate that, despite the high FW content, the microbial community can self-regulate and maintain stability by adjusting to substrate composition, preventing acidification, and sustaining biogas production. Niche overlapping is predicted to be less prevalent with a higher FW content, as microbes take advantage of different substrates without entering into competition. The main metabolic exchanges correlating with the FW content are related to carbohydrate degradation, particularly starch and the heteropolysaccharide pectin. This suggests a form of commensalism arising between species, where hydrolytic microbes benefit from those utilizing the respective degradation products, creating a stable balance of metabolisms. In fact, positive interactions that can be explained by cross-feeding appear to be more evident in HFW and involve a broader set of metabolites. *Methanothrix* sp., the main acetoclastic archaeon in the systems, exemplifies a species likely engaging in a mutualism interaction with *Flexilinea* sp., primarily facilitated by the exchange of amino acids.

Experimental corroboration of these results could improve the stability of the process where a low methane yield is achieved, probably due to the scarce availability of amino acids. Future advancements in metabolic modeling should aim to improve the predictability of AD performance through two primary approaches. The first approach involves refining the structure of GSMMs via pan-genomic analysis and further constraining the model with metabolomic, metatranscriptomic, or environmental data.^53,89^ The second, more sophisticated approach focuses on dynamic modeling techniques. This includes improving the accuracy of extracellular enzyme activity predictions, incorporating kinetic parameters, and integrating real-time data collection from operational digesters. These enhancements will enable the development of more sophisticated models that account for temporal variations in microbial activity and provide actionable insights into optimizing AD systems.

These findings not only contribute to fundamental knowledge in microbial ecology but also offer practical insights for optimizing AD processes, especially concerning the utilization of diverse feedstocks. Accurate models of microbial interactions allow for the precise adjustment of the operational conditions to promote the growth of key microbial populations. For example, by understanding the synergistic relationships between specific bacterial and archaeal species, the process can be fine-tuned to bioaugment the metabolic pathways that drive biogas production. This optimization could reduce the reliance on empirical adjustments, saving time and resources. As we continue to explore and harness microbial communities for environmental and industrial purposes, this study serves as a blueprint for unraveling the complexities of microbial interactions in engineered ecosystems.

## Data Availability

(Supplementary Data S1–S6) MAGs reconstructed, fluxes of the species, intracellular reactions, the script used to add extracellular reactions to the individual models, additional files, and the Python script to perform simulations are available in Zenodo (https://doi.org/10.5281/zenodo.14704579).

## Supporting Information Available

Supplementary methodologies regarding the reactor setup and parameters, genome-scale metabolic model reconstruction, and community flux balance analysis performed and additional results (Figures S1–S9) (PDF)
Additional tables with sample information and biochemical parameters, overview of the feedstock composition, media used for simulations, abundance of the MAGs across the samples, extracellular reactions added to individual GSMMs, and constraints applied to individual GSMMs and exchange fluxes (Tables S1–S10) (XLSX)
