# Supplementary material for: Sustainable Food Waste Management in Anaerobic Digesters: Prediction of the Organic Load Impact by Metagenome-Scale Metabolic Modeling
Source: Environ Sci Technol. 2025 Mar 24;59(13):6659–72. doi: 10.1021/acs.est.4c11180 (PMC11984103; doi:10.1021/acs.est.4c11180)
Supplement: Supplementary file 1 — es4c11180_si_001.pdf [file es4c11180_si_001.pdf]

Sustainable Food Waste Management in Anaerobic Digesters: prediction of organic load impact by Metagenome-Scale Metabolic Modeling

Esteban Orellana[1], Guido Zampieri[1], Nicola De Bernardini[1], Leandro D.Guerrero [2], Leonardo Erijman[2,3], Stefano Campanaro[1*], Laura Treu[1]

1 - Department of Biology, University of Padua, Via U. Bassi 58/B, 35131, Padua, Italy
2 - Instituto de Investigaciones en Ingeniería Genética y Biología Molecular "Dr Héctor N. Torres" (INGEBI-CONICET) Vuelta de Obligado, 2490 - C1428ADN, Buenos Aires, Argentina
3 - Departamento de Fisiología, Biología Molecular y Celular, Facultad de Ciencias Exactas y Naturales. Universidad de Buenos Aires. Intendente Güiraldes, 2160 - C1428EGA, Buenos Aires, Argentina
*Email: stefano.campanaro@unipd.it

Summary

13 Pages
9 Figures

Supplementary Methods

System setup and monitored parameters

To facilitate the creation of genome-scale metabolic models and perform FBA, previous data was selected from literature[1]. Briefly, four laboratory-scale anaerobic reactors (R1-R4) were operated in semi-continuous mode for 161 days at 35 °C. The inoculum used to set up the anaerobic reactors in the laboratory was collected from the anaerobic digester of the municipal treatment plant Norte II of the AySA company, located in San Fernando, Province of Buenos Aires, Argentina. The digester was operated at a mesophilic temperature and with a hydraulic retention time (HRT) of 21 days. The total and volatile solids (TS and VS) of the inoculum were 2,920 and 1,920 mg L^{-1} respectively, while total alkalinity (TA) was 2.71 g $CaCO_3$ L^{-1}, volatile fatty acids (VFA) were 337.50 mg L^{-1} and pH was 7.12. The ratio of primary sludge to activated sludge was approximately 4:1, defined as SS, which was used as a daily substrate for the 4 laboratory reactors. TS and VS of the SS were 4,650 and 3,250 mg L-1, respectively, and Total Kjeldahl nitrogen (TKN) was 2,153 mg L^{-1} (699 mg L^{-1} ammoniacal nitrogen and 1,454 mg L^{-1} organic nitrogen). For the co-substrate, synthetic mixtures were prepared that mimic the typical composition of restaurant food waste. The final synthetic mixture contained 17% total solids (90% volatile). The protein, fat and carbohydrate contents were 3.2%, 0.6% and 12.6% of dry weight. TKN in the feed residue averaged 5.1 g kg^{-1} (Supplementary tables - summary).

Retention time of the solids was 21 days, the feed proportion was initially consistent across all reactors, with each reactor initially receiving the same amount of SS. After 21 days of operation, the feed composition was differentiated by proportions of SS and FW per reactor.

The biogas composition and production were measured daily, while VFA and pH were measured weekly. The CH_4 yield was calculated as the cumulative volume of CH_4 produced per gram of VS added.

Samples were taken during the 161 days of operation in each reactor (**Fig. S1**, **Supplementary Tables S1**).

Genome-scale metabolic models reconstruction

Genome-scale metabolic models (GSMMs) were reconstructed with gapseq[2] from metagenome-assembled genomes (MAGs) with over 90% completeness. MAGs were classified into archaea, Gram-positive bacteria, and Gram-negative bacteria based on taxonomic identification to facilitate the reconstruction. The process followed by gapseq involved several steps: the find step used the parameters -p all and -b 200 to identify all potential metabolic pathways while incorporating Gram stain and domain information for each MAG. This was followed by the find-transport step with the parameter -b 200 to detect transport reactions. The third step, draft, used parameters -u 200 and -l 50 to generate comprehensive draft models. Before continuing with the gap-filling step, draft models were manually modified to integrate extracellular reactions by means of a custom R script (addReactions.R). The script filtered the reactions specific to each model from a data frame, parsing the metabolites and their stoichiometry. The *sybil* and *stringr* libraries were used to manage and manipulate the model files. Finally, the modified models were saved in RDS format, ensuring that each model contained the specified extracellular reactions for subsequent metabolic analysis and simulations.

For each model, a growth medium was generated using the *gapseq medium* module in an anoxic environment with the *-c cpd00007:0* parameter. Subsequently, gap-filling of the models was performed using their corresponding medium. Several model features, such as the number of reactions, metabolites, and dead-end metabolites, were analysed to compare the structural properties of the models using MEMOTE (**Figure S6**). Results on these metrics indicate similarities with other studies[3] indicating an accurate draft GSMM reconstruction.

Finally, extensive literature knowledge was used to set exchange reaction directionality for individual GSMMs on relevant compounds such as methane, acetate and methanol in archaea and pectin and hemicellulose in bacteria (**Supplementary Tables S6**). In this way, correct use of hydrolytic and methanogenic pathways was ensured.

Community flux balance analysis

To analyse metabolite flow, flux balance analysis (FBA) was applied. FBA is a mathematical approach that involves constructing a metabolic network based on a set of stoichiometrically balanced biochemical reactions. This network is then transformed into a mathematical model represented by a matrix (designated as S), where each row corresponds to a metabolite and each column to a reaction. To simulate growth, a biomass reaction is added to the network, representing the set of metabolites required during cell duplication. Exchange reactions are also included to allow the import and export of metabolites between the growing medium and the cells. In a steady state, the flux for each reaction is defined by the equation $S \cdot v = 0$, forming a system of linear equations. To predict the maximum growth rate, an objective function $Z = c^T \cdot v$ is defined, where c is a vector assigning weights to indicate each reaction's contribution to the objective. When optimising a single reaction c is generally a vector of zeros with a single value of 1 at the target reaction's position. Thus, for growth, the objective function becomes $Z = v_{biomass}$, where only the flux through the biomass reaction contributes to the outcome[5].

To simulate microbial community metabolism across samples with different concentrations of food waste, MICOM[4] was employed using cooperative tradeoff FBA (ctFBA) on MAGs with a relative abundance higher than 0.1% for each sample. The ctFBA method integrates both microbial community interactions and individual metabolic capabilities, allowing for a realistic representation of microbial behaviour in mixed cultures. The method is based on the assumption that, in a system with inflow of nutrients and outflow of organic matter, present microbial organisms have to grow to maintain steady-state abundances. In ctFBA, each community member i is associated with a stoichiometric matrix $\boldsymbol{S}_i$, a metabolic reactions flux vector $\boldsymbol{v}_i$, a relative abundance a_i, and a growth rate μ_i. An external compartment represents the environment, where nutrient exchanges between each community member and the environment are represented by $\boldsymbol{v}_i^{ex}$, while the overall exchanges between the entire community and the environment are denoted by $\boldsymbol{v}^m$. These fluxes are bound by community constraints, ensuring biologically feasible predictions as follows:

$$\begin{gathered}\forall \boldsymbol{i}: \\ \boldsymbol{S}_i \boldsymbol{v}_i = 0 \\ \mu_i \geq \mu_i^{min} \\ \boldsymbol{v}_i^{lb} \leq \boldsymbol{v}_i \leq \boldsymbol{v}_i^{ub} \\ \boldsymbol{v}_i^{lb,ex} \leq a_i\, \boldsymbol{v}_i^{ex} \leq \boldsymbol{v}_i^{ub,ex} \\ \boldsymbol{v}^{lb,m} \leq \boldsymbol{v}^m \leq \boldsymbol{v}^{ub,m}\end{gathered}$$

Applying these constraints, the ctFBA framework is established as follows:

$$\min \sum_i \mu_i^2$$

$$\text{such that } \mu_c \geq \alpha \mu_c^{max} \text{ and community constraints}$$

$$\text{where } \mu_c^{max} = \max \mu_c \text{ and } \mu_c = \sum_i a_i \mu_i$$

In this case, μ_c denotes the overall growth rate of the microbial community, while the trade-off parameter α, ranging from 0 (indicating no cooperation) to 1 (indicating full cooperation), adjusts the balance between community cooperation and individual growth. The model first calculates the maximum achievable community growth rate μ_c^{max}, and then minimises the L2 norm of individual taxa growth rates. This process yields unique, balanced growth rates for each taxon that are consistent with both individual growth maximisation and the overall optimization of μ_c, ensuring coordinated growth across community members and realistic predictions of community dynamics. In our case, we assumed a quasi-steady state for microbial growth in the bioreactors due to the long temporal distances between sampling points. An exploratory search for the optimal tradeoff value, controlling the balance between community cooperation and individual organism growth, was conducted with values ranging from 0.4 to 1.0, analogously to previous analyses[4,6]. The optimal tradeoff value was determined by comparing the community model predictions with experimental data, such as CH_4, CO_2 production rates and microbial replication rates derived from CoPTR[7] analysis. Based on these comparisons, a tradeoff of 0.7 was selected, ensuring a balance between cooperative and individual growth that best reflected the experimental results. Additionally, a minimum growth rate of 0.01 was applied to all organisms to avoid unrealistic growth predictions. To limit the space of possible solutions and ensure biologically meaningful flux predictions, the "pfba" (parsimonious flux balance analysis) parameter in MICOM was set to

"True," thus focusing on flux solutions with minimal total flux across the community. This choice was based on the assumption that microbes invest minimal resources for growth and do not over-produce unnecessary enzymes. Empirically, this has been shown to work well in isolated organisms[8].

The input to these simulations included both sewage sludge (SS) and food waste (FW) media composition (detailed in **Supplementary Tables S2**) as well as individual microbial abundance data for each sample. Volatile fatty acids, which play a critical role in anaerobic digestion processes, were used as additional constraints on the communities. Furthermore, several key metabolic reactions were manually curated guided by reference on isolated organisms (as detailed in **Supplementary Tables S6**) The use of acetate by acetoclastic archaea was verified across all samples. Uptake of several monosaccharides such as glucose, glycerol, and D-glyceraldehyde, along with polysaccharides, was constrained in the archaea to prevent inconsistent competition with fermenters. This approach ensured that the model accurately represented microbial metabolism under the experimental conditions, enabling reliable predictions of biogas production. Output fluxes were normalised by the MAG's relative abundance.

Supplementary Results and Discussion

Amino acids exchange

The primary organism responsible for AA production, *Ancrocorticia* sp. 70, can synthesise L-Lysine from aspartate or L-homoserine, with a higher flux observed in the case of aspartate. *Methanothrix* sp. 1 showed higher levels of consumption of L-Alanine, L-Aspartate, L-Asparagine and L-Threonine than other archaea, which is possibly correlated with the highest CH_4 production (spearman correlation coefficient r=0.45 and r=0.55 respectively, p-value < $1e^{-3}$). It utilises L-alanine through the action of L-alanine dehydrogenase, a process that converts it into pyruvate and NH_3 (ModelSeed: rxn00278).

A modest Spearman correlation was also found between the archaeon abundance and the FW (r=0.45, p-value < $1e^{-3}$), however, the correlation significantly increased to 0.80 when considering the activity of 12 out of the 14 reactions where L-Aspartate participates. This difference suggests that activities related to some compounds, like L-Aspartate, provides a more nuanced understanding of AA association with the varying feedstock conditions. In the case of asparagine, it was used for the production of L-Aspartate (ModelSeed: rxn00342), while L-Threonine is used for the synthesis of 2-Amino-acetoacetate, which is deacetylated to form glycine and Acetyl-CoA. L-cysteine can cause an increase in CH_4 production through the acetoclastic pathway by activating the conversion of glucose to acetic acid[9]. However, the addition of the co-substrate resulted in the accumulation of L-cysteine, indicating that its contribution to CH_4 production could not be as important as expected.

Metabolites Exchange and Extracellular Reactions

Correlation analysis between total fluxes of extracellular reactions in each sample and FW content identified 58 reactions with positive correlations (r > 0.4, p-value < 0.005, **Supplementary Tables S8**). The reaction with the highest correlation involves an exo-polygalacturonase, which degrades pectin to produce D-galacturonate from pectate (KEGG Reaction: R04320 - rxn06064), as discussed in the main text. Several sucrose glucohydrolases also showed high correlations (r > 0.77). The primary organism involved in

this activity is from the family *Ruminococcaceae* (*Ruminococcaceae* sp. 47). Members of this family are important in starch degradation in the human gut[10], and metabolic modelling of one species showed sucrose consumption.

Correlations between flux exchanges and FW concentration revealed 162 metabolites with absolute correlations higher than 0.5 (p-value < 0.0005, **Supplementary Tables S9**), with xylitol and xylose showing the highest correlations. These compounds are obtained from xylan degradation, and the negative flux indicates monomer production, meaning there is zero flux in samples without FW due to the absence of the polymer. The addition of FW introduces xylan, which is degraded, releasing xylose and xylitol for cellular use. This reaction is primarily performed by *Bacteroides* sp. 26. Metabolic modelling of a species from the *Bacteroides* genus showed xylan consumption[11], supporting these results.

Several polymers and disaccharides, such as starch, sucrose, and digalacturonate, correlate negatively with FW, indicating their abundance increases with more FW. Some compounds result from degradation, either through secretion or hydrolysis. A particular reaction involves lipase degradation, liberating glycerol, and is carried out by *Smithellaceae* sp. 23, which is more abundant in medium food waste concentrations. Moreover, a production of galactose leads to a high correlation with FW (r=0.82). This metabolite is produced by the enzyme lactose galactohydrolase, which was carried by mostly two bacteria from the Bacteroidales order (*Bacteroidales* sp. 31 and *Bacteroidales* sp. 111). Another enzyme, melibiose galactohydrolase, produces galactose from melibiose dissociation and is performed by Bacteroides sp. 25. This enzyme is part of *Bacteroides thetaiotaomicron*[12], supporting the utilization of this substrate by *Bacteroides* sp. Fluxes of extracellular reactions for each model are reported in **Supplementary Tables S10**.

Supplementary Figures

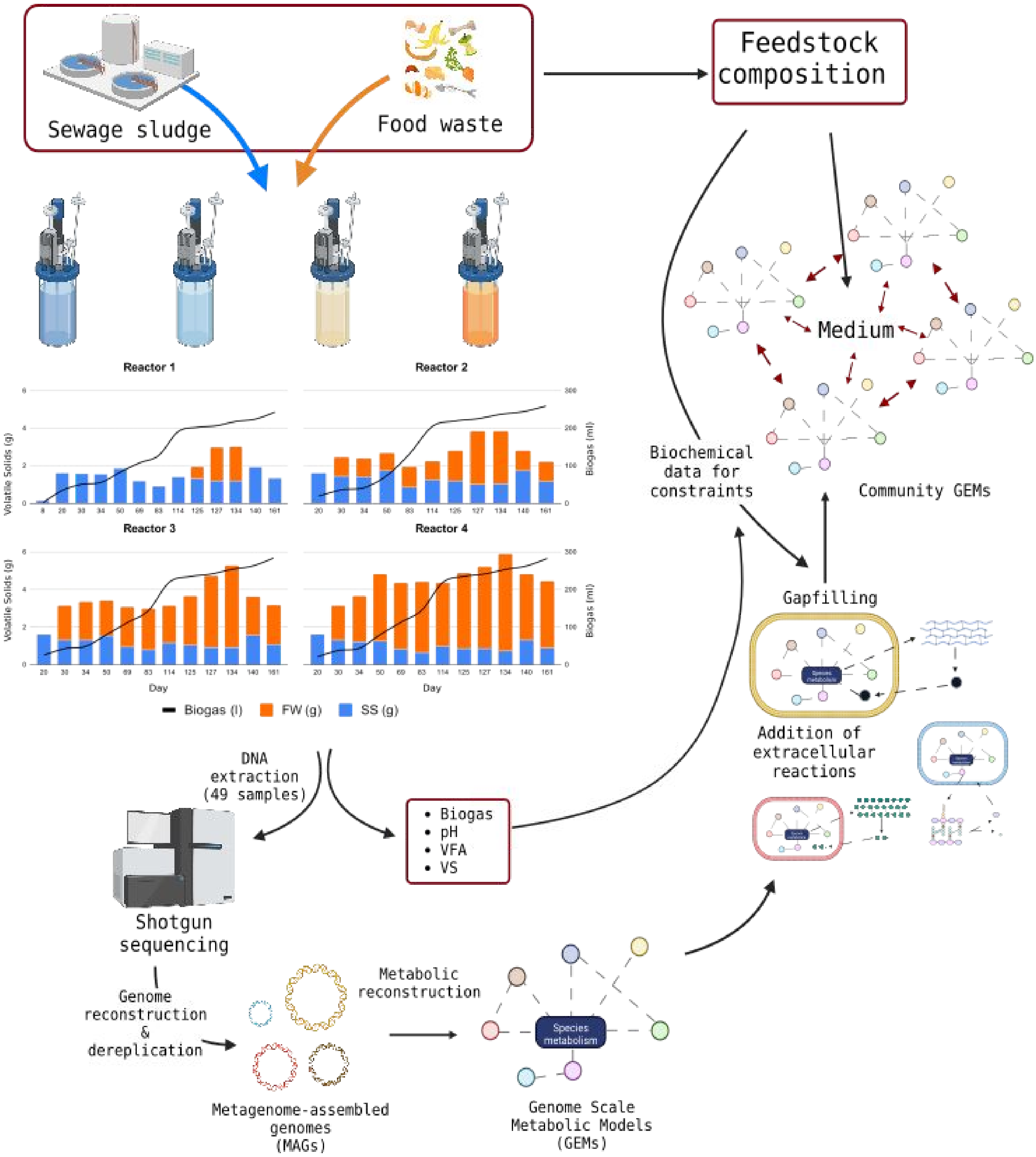

Figure S1: Experimental design. Four lab-scale reactors were fed with different proportions of sewage sludge taken from a municipal wastewater treatment plant (blue) and food waste (orange). Metagenome-scale metabolic modelling was performed with MAGs from forty-nine samples and biochemical parameters for the gapfilling and constraint of the models.

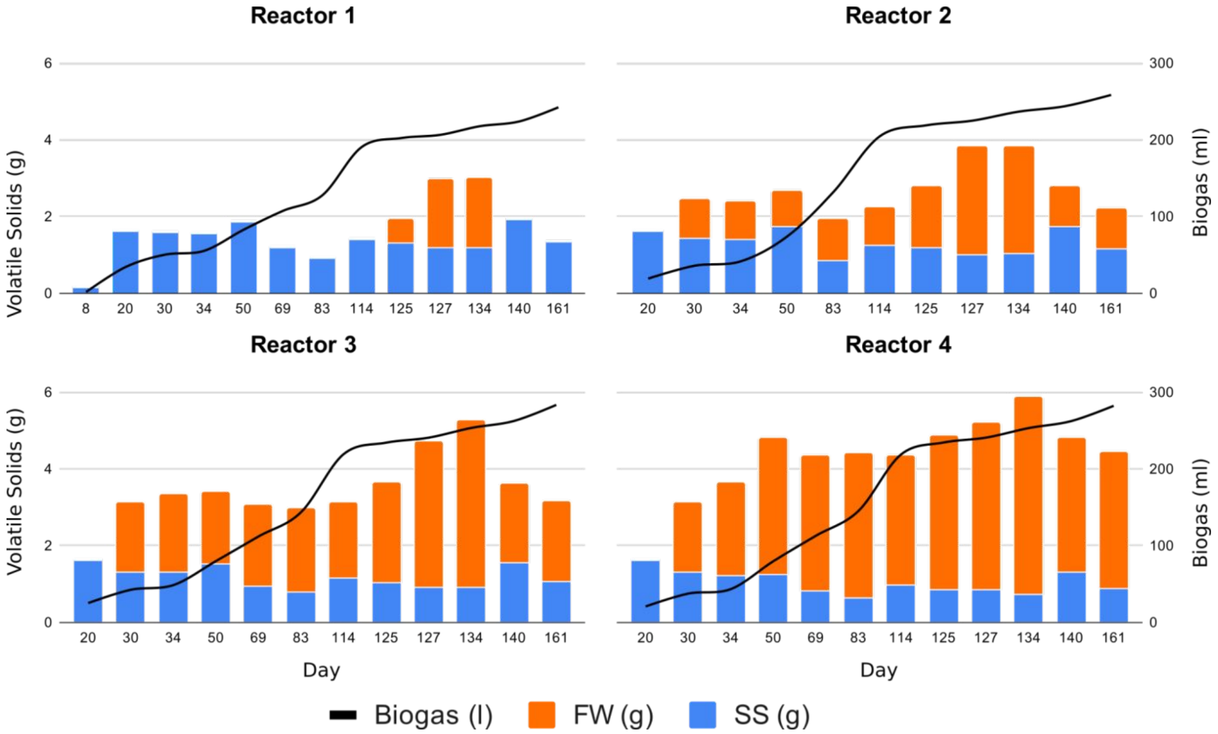

Figure S2: Volatile solids fed to the digesters and biogas production. Blue bars indicate sewage sludge and orange food waste added to the reactors. Biogas is represented by black lines.

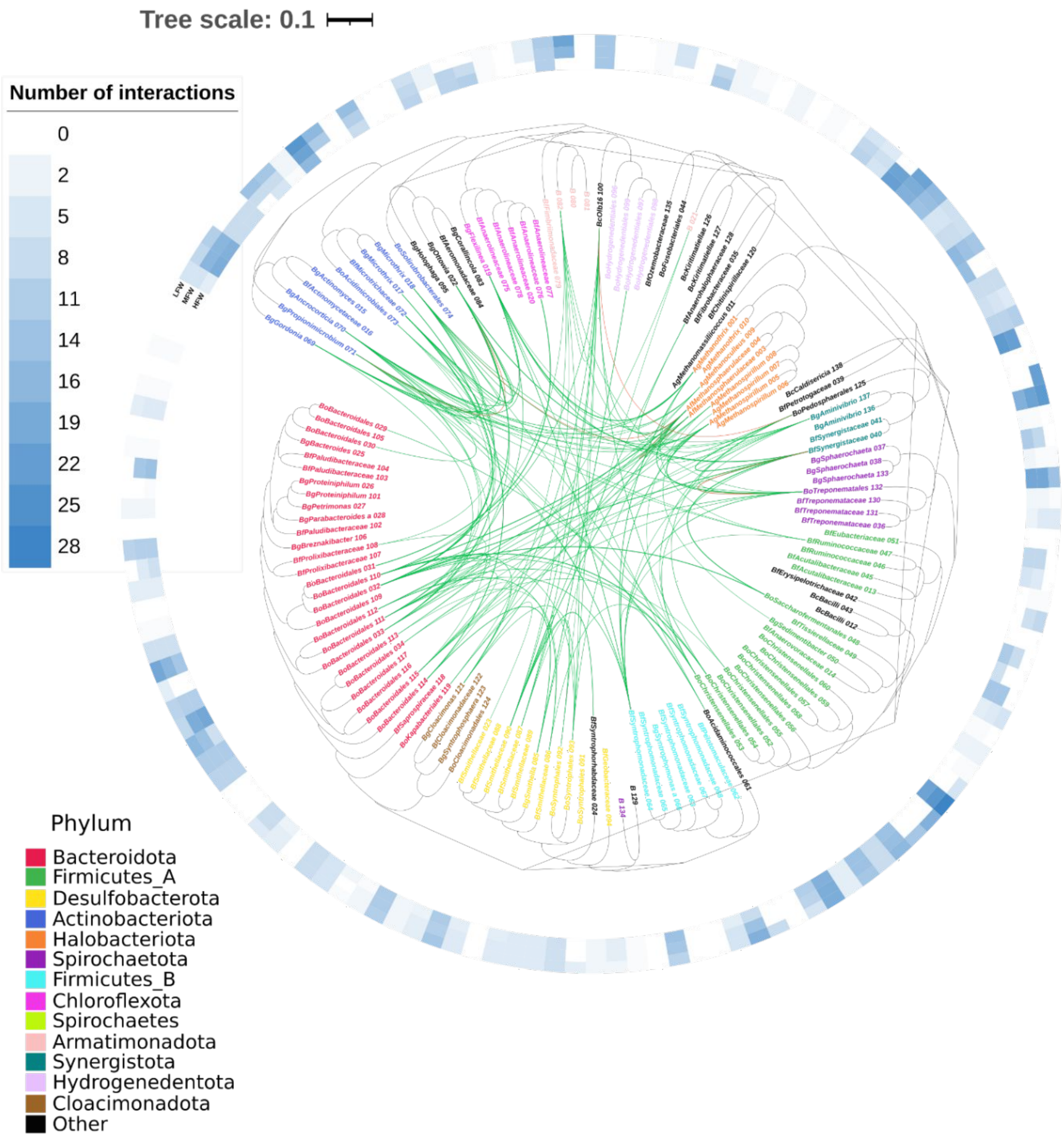

Figure S3: Phylogenetic tree of the 138 MAGs used in this study. Blue boxes indicate the number of significant interactions detected for each MAG. Inner colours represent the taxonomic classification. Inner green lines indicate mutualistic interactions and red lines competition between organisms.

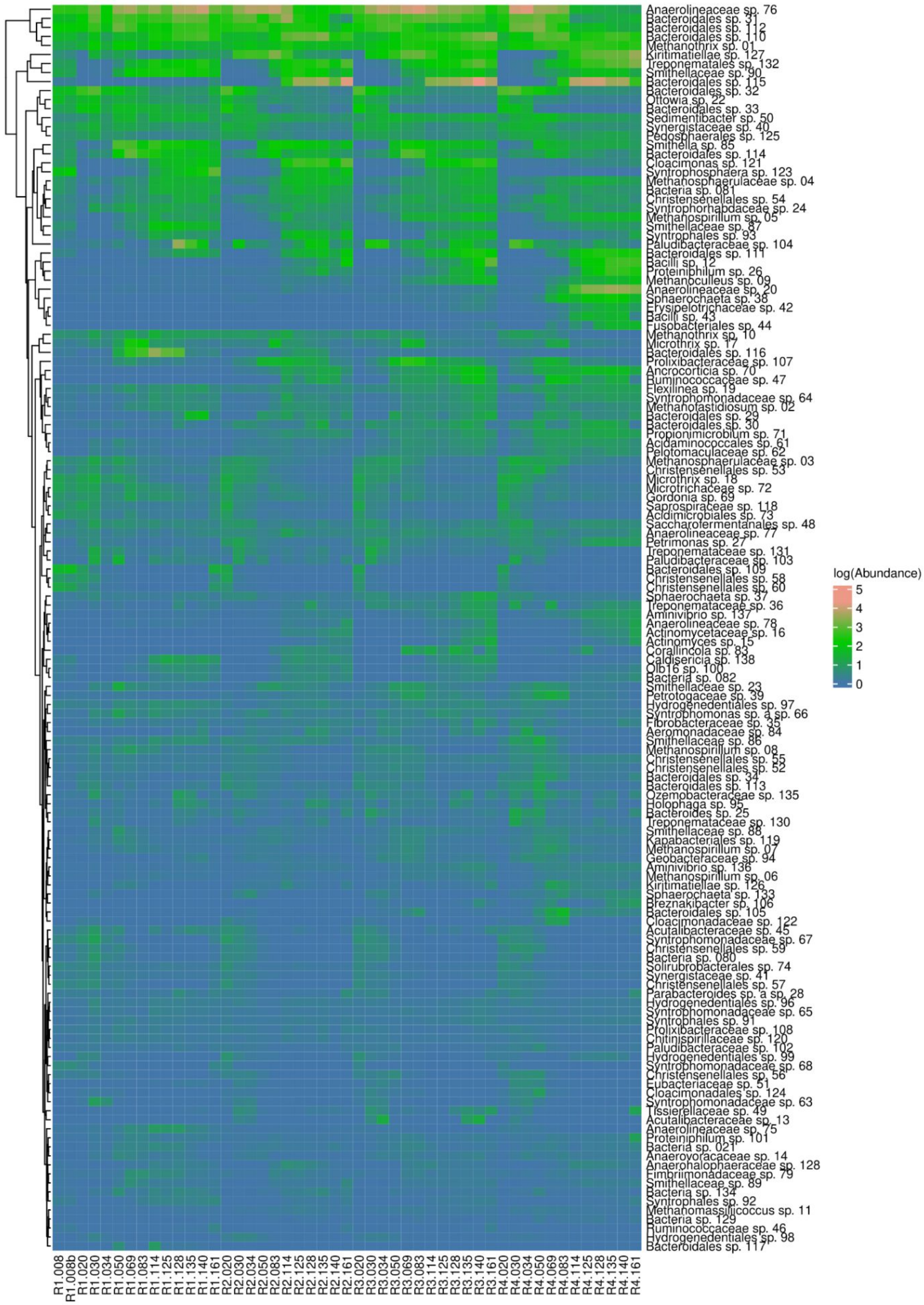

Figure S4: Heatmap showing the logarithmic representation of the abundance of MAGs across 49 samples. Abundance values were calculated using CoverM (https://github.com/wwood/CoverM) with the genome flag and the mean method.

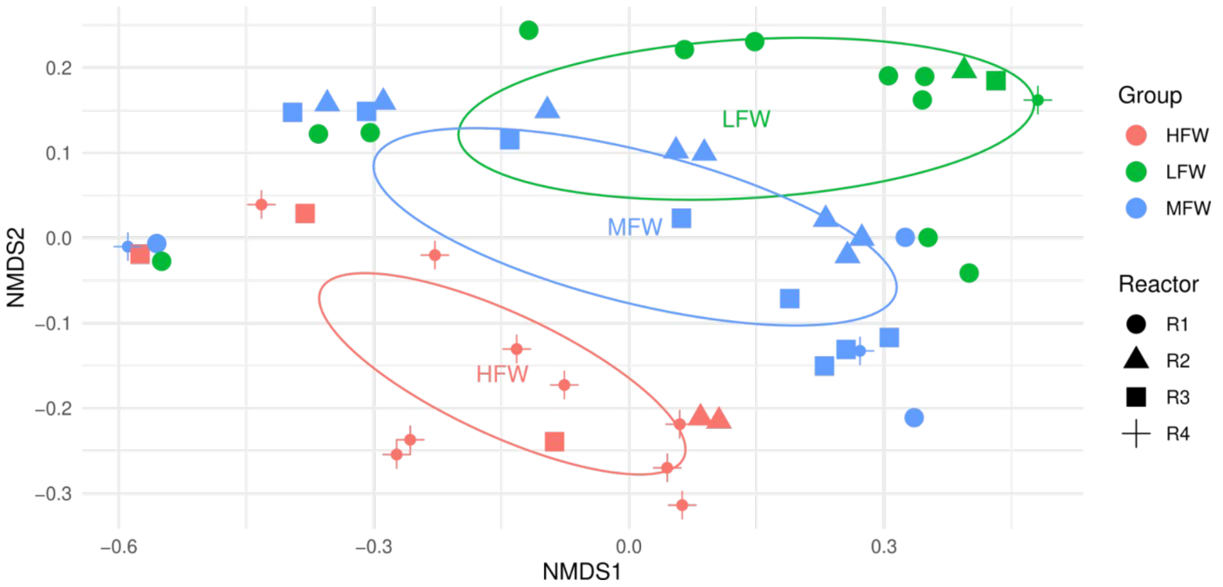

Figure S5: Non metric multidimensional scaling (NMDS) plot showing the distribution of MAGs based on their abundance across samples. The NMDS analysis was performed using the vegan package in R, with the abundance matrix transformed using the Bray-Curtis distance.

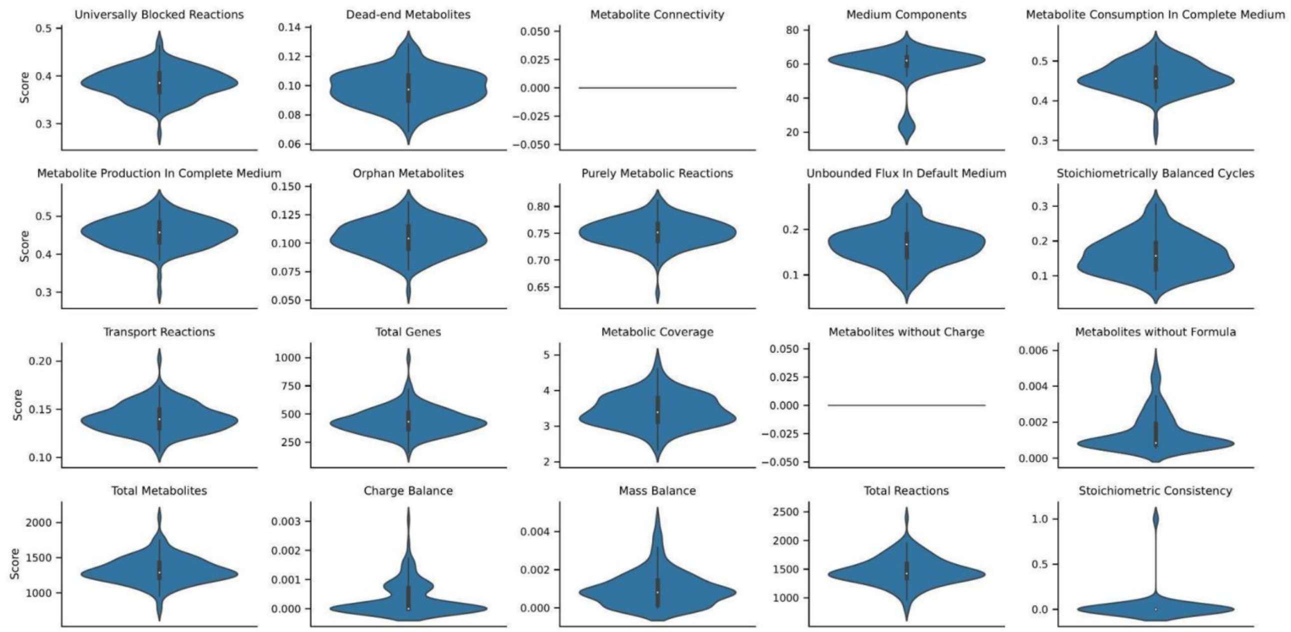

Figure S6: Memote results for all the models.

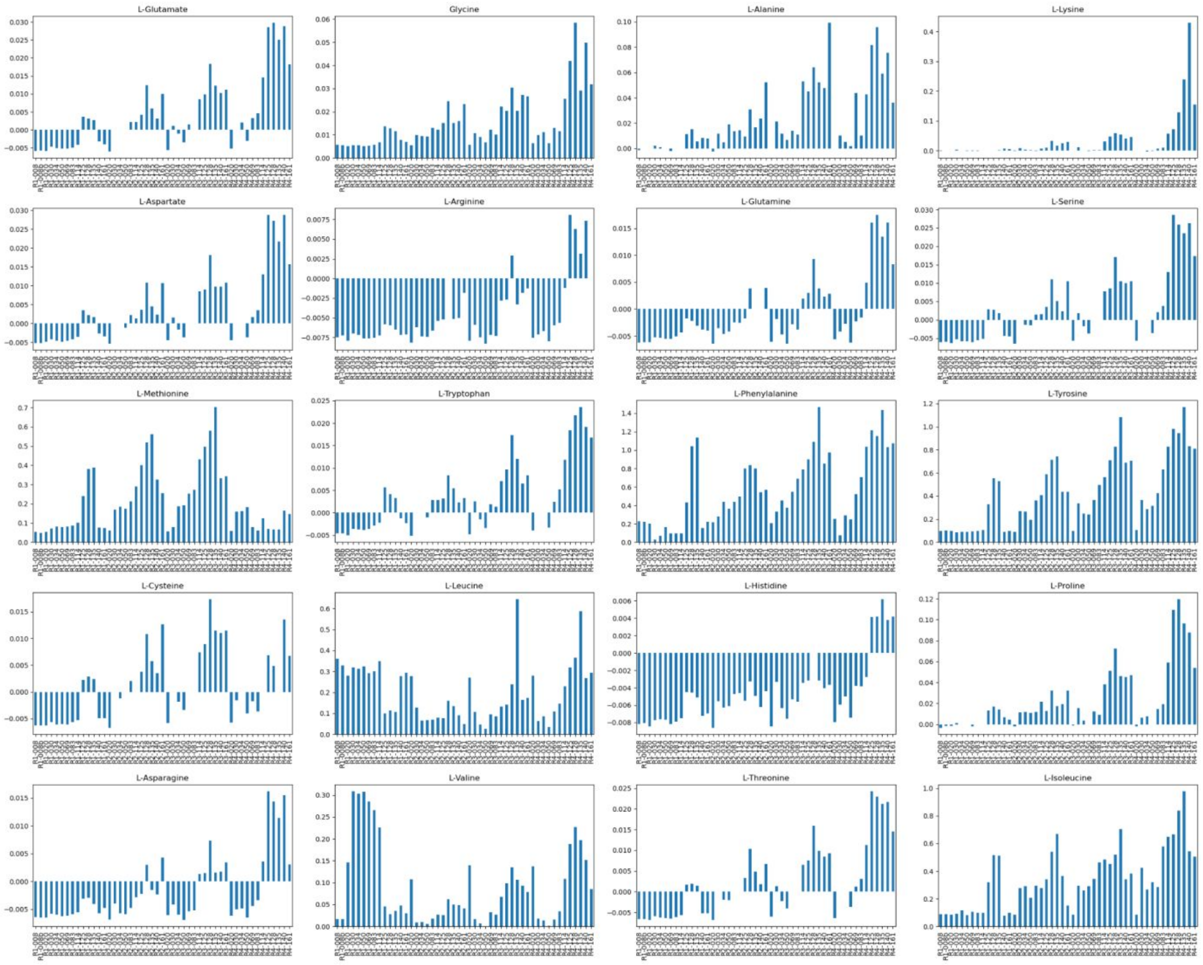

Figure S7: Amino acid fluxes. Each box represents the fluxes of a single amino acid between the medium and the organisms. Vertical axis represents flux in mmol of the AA * g VS^{-1} * h^{-1}.

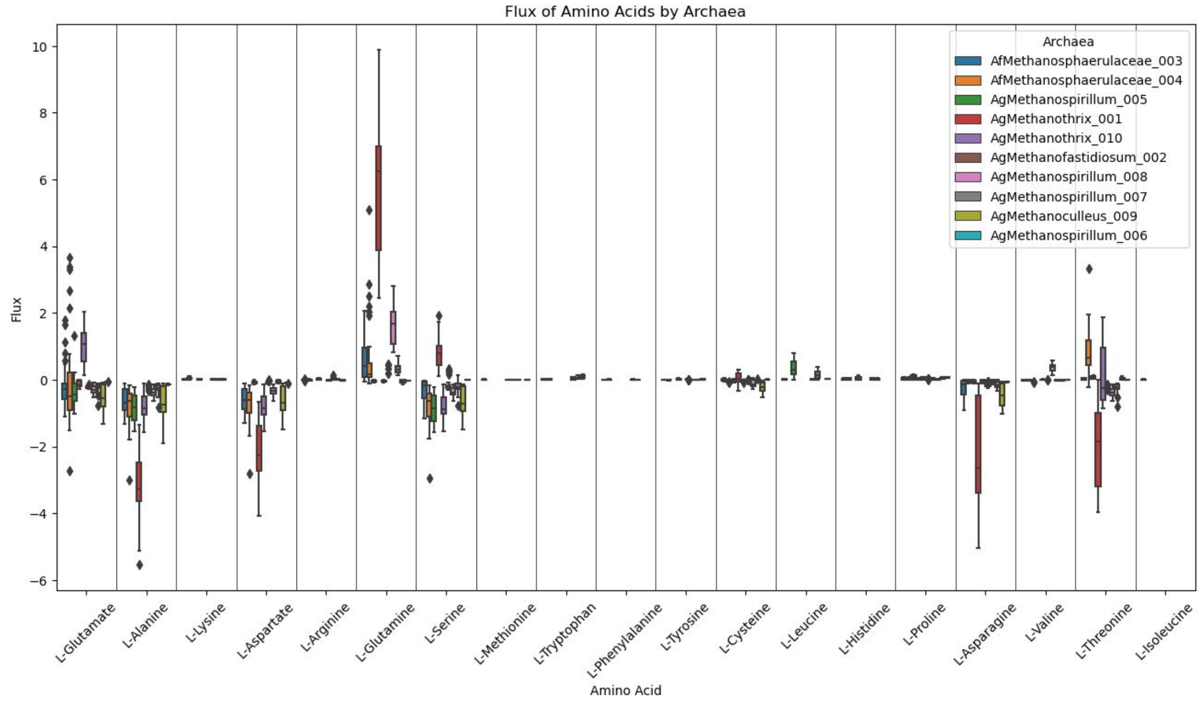

Figure S8: Amino acid exchange for each organism. Distribution of AA exchange for each organism through the samples. Vertical axis represents flux in mmol of the AA * g VS^{-1} * h^{-1}.

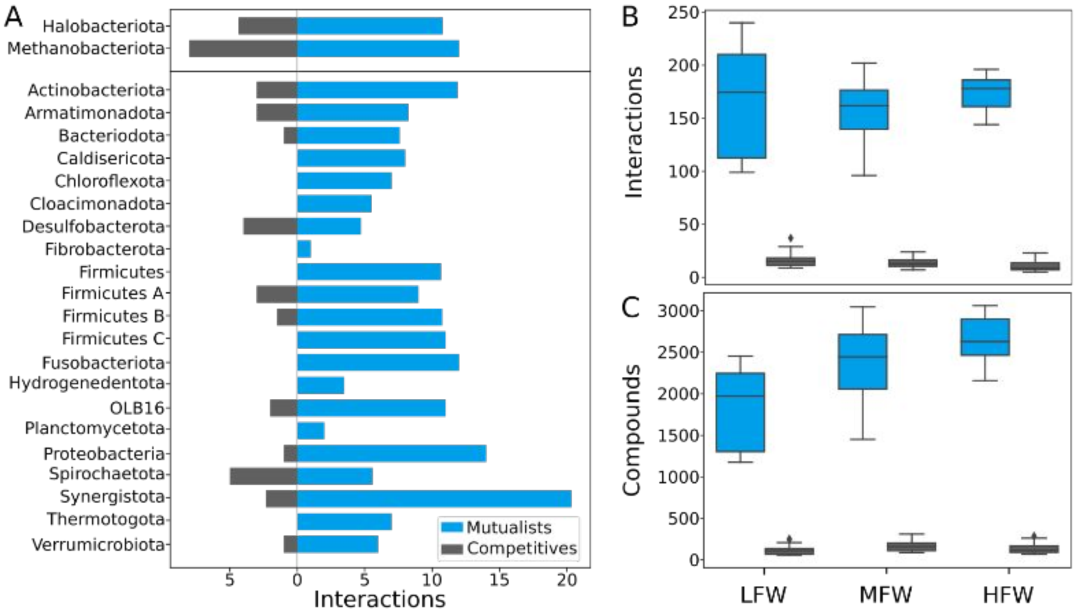

Figure S9: Interaction between species. A - Mutualistic (blue bar) and competitive (gray bar) interactions for each phyla. B - Comparison of each type of interactions in low-food waste samples (LFW), medium FW (MFW) and high FW (HFW) concentrations. C - Total number of metabolites (Compounds) shared in each type of interaction in the different samples according to the FW concentration.

References

(1) Orellana, E.; Davies-Sala, C.; Guerrero, L. D.; Vardé, I.; Altina, M.; Lorenzo, M. C.; Figuerola, E. L.; Pontiggia, R. M.; Erijman, L. Microbiome Network Analysis of Co-Occurrence Patterns in Anaerobic Co-Digestion of Sewage Sludge and Food Waste. *Water Sci. Technol.* **2019**, *79* (10), 1956–1965. https://doi.org/10.2166/wst.2019.194.

(2) Zimmermann, J.; Kaleta, C.; Waschina, S. Gapseq: Informed Prediction of Bacterial Metabolic Pathways and Reconstruction of Accurate Metabolic Models. *Genome Biol.* **2021**, *22* (1), 81. https://doi.org/10.1186/s13059-021-02295-1.

(3) Lieven, C.; Beber, M. E.; Olivier, B. G.; Bergmann, F. T.; Ataman, M.; Babaei, P.; Bartell, J. A.; Blank, L. M.; Chauhan, S.; Correia, K.; Diener, C.; Dräger, A.; Ebert, B. E.; Edirisinghe, J. N.; Faria, J. P.; Feist, A. M.; Fengos, G.; Fleming, R. M. T.; García-Jiménez, B.; Hatzimanikatis, V.; van Helvoirt, W.; Henry, C. S.; Hermjakob, H.; Herrgård, M. J.; Kaafarani, A.; Kim, H. U.; King, Z.; Klamt, S.; Klipp, E.; Koehorst, J. J.; König, M.; Lakshmanan, M.; Lee, D.-Y.; Lee, S. Y.; Lee, S.; Lewis, N. E.; Liu, F.; Ma, H.; Machado, D.; Mahadevan, R.; Maia, P.; Mardinoglu, A.; Medlock, G. L.; Monk, J. M.; Nielsen, J.; Nielsen, L. K.; Nogales, J.; Nookaew, I.; Palsson, B. O.; Papin, J. A.; Patil, K. R.; Poolman, M.; Price, N. D.; Resendis-Antonio, O.; Richelle, A.; Rocha, I.; Sánchez, B. J.; Schaap, P. J.; Malik Sheriff, R. S.; Shoaie, S.; Sonnenschein, N.; Teusink, B.; Vilaça, P.; Vik, J. O.; Wodke, J. A. H.; Xavier, J. C.; Yuan, Q.; Zakhartsev, M.; Zhang, C. MEMOTE for Standardized Genome-Scale Metabolic Model Testing. *Nat. Biotechnol.* **2020**, *38* (3), 272–276. https://doi.org/10.1038/s41587-020-0446-y.

(4) Diener, C.; Gibbons, S. M.; Resendis-Antonio, O. MICOM: Metagenome-Scale Modeling To Infer Metabolic Interactions in the Gut Microbiota. *mSystems 5* (1), e00606-19.

https://doi.org/10.1128/mSystems.00606-19.
(5) Orth, J. D.; Thiele, I.; Palsson, B. O. What Is Flux Balance Analysis? *Nat. Biotechnol.* **2010**, *28* (3), 245–248. https://doi.org/10.1038/nbt.1614.
(6) Zampieri, G.; Campanaro, S.; Angione, C.; Treu, L. Metatranscriptomics-Guided Genome-Scale Metabolic Modeling of Microbial Communities. *Cell Rep. Methods* **2023**, *3* (1).
(7) Joseph, T. A.; Chlenski, P.; Litman, A.; Korem, T.; Pe'er, I. Accurate and Robust Inference of Microbial Growth Dynamics from Metagenomic Sequencing Reveals Personalized Growth Rates. *Genome Res.* **2022**, *32* (3), 558. https://doi.org/10.1101/gr.275533.121.
(8) Machado, D.; Herrgård, M. Systematic Evaluation of Methods for Integration of Transcriptomic Data into Constraint-Based Models of Metabolism. *PLOS Comput. Biol.* **2014**, *10* (4), e1003580. https://doi.org/10.1371/journal.pcbi.1003580.
(9) Paudel, Y. P.; Lin, C.; Shen, Z.; Qin, W. Characterization of Pectin Depolymerising Exo Polygalacturonase by Bacillus Sp. HD2 Isolated from the Gut of Apis Mellifera L. *Microbiol. Discov.* **2015**, *3* (1), 1–8.
(10) Kim, Y.-J.; Jung, D.-H.; Park, C.-S. Important Roles of Ruminococcaceae in the Human Intestine for Resistant Starch Utilization. *Food Sci. Biotechnol.* **2024**, *33* (9), 2009–2019. https://doi.org/10.1007/s10068-024-01621-0.
(11) Neal, M.; Thiruppathy, D.; Zengler, K. Genome-Scale Metabolic Modeling of the Human Gut Bacterium Bacteroides Fragilis Strain 638R. *PLOS Comput. Biol.* **2023**, *19* (10), e1011594. https://doi.org/10.1371/journal.pcbi.1011594.
(12) Shin, Y.-J.; Woo, S.-H.; Jeong, H.-M.; Kim, J.-S.; Ko, D.-S.; Jeong, D.-W.; Lee, J.-H.; Shim, J.-H. Characterization of Novel α-Galactosidase in Glycohydrolase Family 97 from *Bacteroides Thetaiotaomicron* and Its Immobilization for Industrial Application. *Int. J. Biol. Macromol.* **2020**, *152*, 727–734. https://doi.org/10.1016/j.ijbiomac.2020.02.232.
